# Estimating under-five mortality attributable to fine particulate matter brought by wind-blown dust in 100 low- and middle-income countries in 2000–2017

**DOI:** 10.1093/nsr/nwaf279

**Published:** 2025-07-09

**Authors:** Ruohan Wang, Hong Lu, Ning Kang, Mingkun Tong, Jianyu Deng, Xueqiu Ni, Pengfei Li, Tao Xue, Tong Zhu

**Affiliations:** Institute of Reproductive and Child Health / National Health Commission Key Laboratory of Reproductive Health and Department of Epidemiology and Biostatistics / Ministry of Education Key Laboratory of Epidemiology of Major Diseases (PKU), School of Public Health, Peking University Health Science Centre, Beijing 100191, China; Institute of Reproductive and Child Health / National Health Commission Key Laboratory of Reproductive Health and Department of Epidemiology and Biostatistics / Ministry of Education Key Laboratory of Epidemiology of Major Diseases (PKU), School of Public Health, Peking University Health Science Centre, Beijing 100191, China; Institute of Reproductive and Child Health / National Health Commission Key Laboratory of Reproductive Health and Department of Epidemiology and Biostatistics / Ministry of Education Key Laboratory of Epidemiology of Major Diseases (PKU), School of Public Health, Peking University Health Science Centre, Beijing 100191, China; Institute of Reproductive and Child Health / National Health Commission Key Laboratory of Reproductive Health and Department of Epidemiology and Biostatistics / Ministry of Education Key Laboratory of Epidemiology of Major Diseases (PKU), School of Public Health, Peking University Health Science Centre, Beijing 100191, China; Institute of Reproductive and Child Health / National Health Commission Key Laboratory of Reproductive Health and Department of Epidemiology and Biostatistics / Ministry of Education Key Laboratory of Epidemiology of Major Diseases (PKU), School of Public Health, Peking University Health Science Centre, Beijing 100191, China; Institute of Reproductive and Child Health / National Health Commission Key Laboratory of Reproductive Health and Department of Epidemiology and Biostatistics / Ministry of Education Key Laboratory of Epidemiology of Major Diseases (PKU), School of Public Health, Peking University Health Science Centre, Beijing 100191, China; Institute of Medical Technology, Peking University Health Science Centre, Beijing 100191, China; Advanced Institute of Information Technology, Peking University, Hangzhou 311215, China; Institute of Reproductive and Child Health / National Health Commission Key Laboratory of Reproductive Health and Department of Epidemiology and Biostatistics / Ministry of Education Key Laboratory of Epidemiology of Major Diseases (PKU), School of Public Health, Peking University Health Science Centre, Beijing 100191, China; Advanced Institute of Information Technology, Peking University, Hangzhou 311215, China; State Environmental Protection Key Laboratory of Atmospheric Exposure and Health Risk Management, Center for Environment and Health, Peking University, Beijing 100871, China; State Environmental Protection Key Laboratory of Atmospheric Exposure and Health Risk Management, Center for Environment and Health, Peking University, Beijing 100871, China; College of Environmental Sciences and Engineering, Peking University, Beijing 100871, China

**Keywords:** particulate matter, wind-blown dust, under-five mortality, risk assessment

## Abstract

Wind-blown dust (dust PM_2.5_) is a major contributor to fine particulate matter (PM_2.5_) in low- and middle-income countries (LMICs), yet its impact on under-five mortality (U5M) remains underexplored. In particular, due to the lack of an exposure-response function (ERF) focusing on dust PM_2.5_, the relevant burden is evaluated based on pre-established ERFs for total PM_2.5_ mass. Our study aimed to evaluate the association between long-term dust PM_2.5_ exposure and U5M, estimating the attributable mortality burden in LMICs. Using high-resolution PM_2.5_ mass-maps, well-validated dust ratio data, and 125 demographic and health surveys, we applied a fixed-effects Cox model to examine the association between life-course dust PM_2.5_ exposure and the survival status of 1 411 851 children from 53 LMICs. Subsequently, we developed a nonlinear ERF by integrating the marginal effects of within-strata exposure variation, and extrapolated this function to estimate the U5M burden attributable to dust PM_2.5_ across 100 LMICs, comparing results with two existing ERFs for total PM_2.5_. Each 10-μg/m³ increase in dust PM_2.5_ exposure was associated with a 7.13% (95% confidence interval [CI]: 4.54–9.78) increase in U5M risk. The ERF indicated no threshold effect at low concentrations and a steeper slope at higher levels. Based on this function, we estimated that dust PM_2.5_ contributed to ∼1.74 million, 1.30 million, and 1.07 million U5Ms in 2000, 2010, and 2017, respectively. Notably, these estimates exceeded those derived from pre-established ERFs for total PM_2.5_ mass in most countries. Our findings underscore the significant contribution of dust PM_2.5_ to the U5M burden and emphasize the importance of early-warning systems to effectively safeguard child health.

## Teaser

Focusing on the ternary hydrides, the new hope of Room-Temperature Superconductivity, this perspective delves into the research background, highlights current challenges, and illuminates promising avenues for future studies.

## INTRODUCTION

Outdoor air pollution poses a significant threat to the health and wellbeing of children [1–3]. According to the 2019 Global Burden of Disease (GBD) study [4], long-term exposure to ambient particulate matter (PM) has resulted in 233 454 (95% confidence interval [CI]: 161 412–318 743) premature deaths among children under the age of 5 years (i.e. under-five mortality [U5M]), with 99.4% occurring in low- and middle-income countries (LMICs). Due to their immature organs, lower metabolic capacity, and weaker immune system, children are more vulnerable to the harmful effects of air pollutants, particularly particles with a diameter <2.5 μm (fine particulate matter; PM_2.5_) [5–7].

The toxicity of PM_2.5_ depends on its source and chemical composition [8,9]. However, previous assessments of disease burden associated with PM_2.5_ pollution often relied on exposure–response functions that implicitly assumed all fine particles are equally toxic [4,10–12]. Therefore, specific exposure–response functions for dust, the leading contributor to PM_2.5_ mass in some LMICs, such as Egypt and Nigeria, have not been comprehensively evaluated [8,13]. Dust pollution is predominantly influenced by natural factors, such as deserts, topography, atmospheric circulation, and local rainfall, which can be affected by climate change [14,15]. While previous epidemiological studies have identified a positive association between early-life exposure to dust PM_2.5_ and child mortality, the health effects vary depending on the study region and age group of the children [16–18]. In the latest version of the Global Air Quality Guidelines [19], for the first time, the World Health Organization provides qualitative statements on good practices for managing PM_2.5_ particles originating from wind-blown dust (hereafter referred to as dust PM_2.5_), highlighting their potential health risks. However, due to insufficient evidence of the health effects of dust PM_2.5_, formulating relevant air quality guidelines is challenging.

Although previous studies have estimated the mortality burden attributable to ambient PM_2.5_ generated from various sources, including dust PM_2.5_ [8], based on pre-established exposure–response functions, recent findings suggest that these estimates can be inaccurate. An increasing number of studies have demonstrated that the health effects of the total PM_2.5_ mass differ from those of dust PM_2.5_. For instance, a study from Africa estimated that a 10-μg/m^3^ increase in ambient dust PM_2.5_ was associated with a 24% (95% CI: 10–35) increase in infant mortality [20], whereas the same team reported that the risk increased by 9% (95% CI: 4–14) with a similar increase in the total PM_2.5_ mass [21]. However, with regard to the dust-specific exposure–response function based on epidemiological studies, a knowledge gap exists regarding the spatial coverage of the study population, nonlinearity of the function, and threshold of the effect (also known as the theoretical minimum risk exposure level [TMREL]). Considering the vulnerability of children, multicentre epidemiological studies conducted in LMICs are needed to develop a nonlinear exposure–response function between dust PM_2.5_ and U5M.

In this large-scale multicentre study, we evaluated the association between U5M and long-term exposure to dust PM_2.5_, identified the effect threshold, and developed a dust-specific exposure–response function. Additionally, we estimated the attributable mortality burden across 100 LMICs and identified hotspot countries. To validate our estimates, we compared them to attributable burdens generated by pre-established exposure–response functions.

## RESULTS

### Descriptive statistics

To investigate the association between dust PM_2.5_ exposure and U5M, we analysed the survival status of 1 411 851 children from 2525 sampling strata across 53 LMICs. Of these children, 721 335 (51.1%) were boys. During a follow-up period of 3.28 million person-years, 77 067 (5.5%) children died before reaching the age of 5 years. Compared to the control group (mean age = 29.14 ± 17.30 months), children in the case group (mean age = 5.93 ± 9.44 months) were exposed to higher levels of dust PM_2.5_ pollution (23.66 vs. 19.27 μg/m^3^). Furthermore, characteristics associated with the case group included a higher percentage of boys, residence in rural areas, lack of breastfeeding, absence of insurance, and poor household hygiene conditions (Table S3).

**Figure 1.**
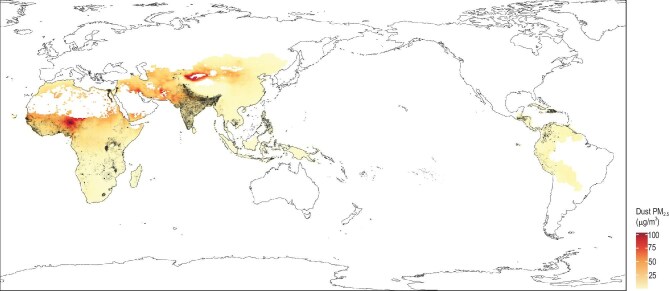
Study domain for risk assessment (grey polygons, 100 LMICs), geographic locations of samples (black dots, obtained from 53 LMICs), and long-term average concentration of dust PM_2.5_ in human settlements (background colours) during 2000–2017. Review drawing number: GS 京 (2025) 1073号.

Figure 1 presents the spatial distribution of study samples and dust PM_2.5_ exposure levels across the risk assessment domain (100 LMICs). The sampled regions cover a wide range of dust PM_2.5_ concentrations, ranging from high-pollution areas in sub-Saharan Africa and Central Asia to regions with lower concentrations such as Southeast Asia, Central Africa (Congo Basin), Latin America, and the Caribbean. Among all countries, Niger exhibited the most severe dust pollution, followed by Nigeria and Mauritania.

### Linear associations

**Figure 2.**
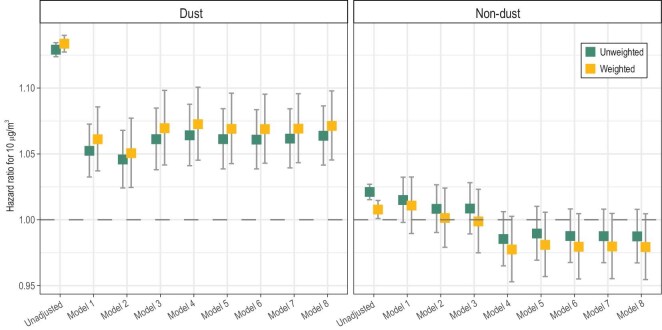
Linear associations between PM_2.5_ exposure (dust and non-dust) and U5M estimated by various models. Model 1: initial model adjusted for the fixed effect of strata; Model 2: Model 1 plus adjustment for seasonal trend (interaction between the climate zone of the survey location and the seasonal child mortality); Model 3: Model 2 plus environmental variables (dust or non-dust components in PM_2.5_ and temperature); Model 4: Model 3 plus demographic characteristics (sex and breastfeeding status); Model 5: Model 4 plus pregnancy-related variables (caesarean section, place of delivery, antenatal care attendance, singleton birth or not, and nulliparous or not); Model 6: Model 5 plus reproductive history-related variables (maternal age and interpregnancy interval); Model 7: Model 6 plus maternal features (maternal body mass index and employment status); Model 8: Model 7 plus household features (sex and age of household head, source of drinking water, and types of toilet and cooking energy).

We used various models to estimate the linear associations of life-course exposure to dust or PM_2.5_ originating from other sources (non-dust PM_2.5_) and U5M (Fig. 2). Notably, the estimated effects for dust PM_2.5_ consistently exhibited statistical significance. Except for the adjustment for fixed effects, the inclusion of seasonal trends, environmental factors, or other demographic characteristics did not significantly influence the estimated effects. Additionally, the results remained relatively stable when employing DHS sampling weights. In the fully adjusted and weighted models, a 10-μg/m^3^ increase in life-course exposure to dust PM_2.5_ was associated with a 7.13% (95% CI: 4.54–9.78) increase in the U5M risk. Conversely, the effects of PM_2.5_ originating from other sources were not significant (hazard ratio = 0.98, 95% CI: 0.95–1.00).

Furthermore, we conducted a sensitivity analysis based on exposure time windows, including prenatal exposure, life-course exposure, and exposure across various time lags, ranging from 1 to 12 months before the occurrence of outcomes (Fig. S1). Compared to postnatal exposure, the estimated effects were smaller for dust PM_2.5_ exposure during pregnancy (hazard ratio = 1.03, 95% CI: 1.01–1.05). Additionally, the risk of dust PM_2.5_ is highest at a lag of 1–8 months (with non-dust adjustment, hazard ratio = 1.28, 95% CI: 1.24–1.31). In this study, the life course served as the primary time-window in our main model to fully capture the risks associated with exposure.

### Nonlinear association

**Figure 3.**
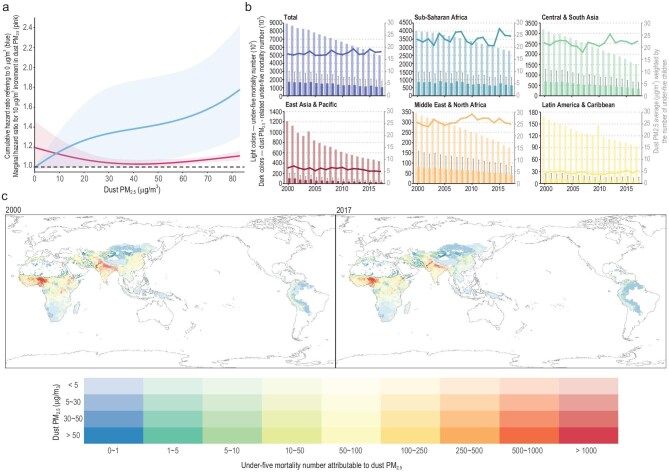
Burden assessment of life-course dust PM_2.5_ exposure on U5M in 100 LMICs. Panel (a) presents the nonlinear exposure–response function for life-course dust PM_2.5_ exposure and U5M. The blue line and corresponding ribbon indicate the hazard ratio of U5M and its 95% CI, respectively, according to dust PM_2.5_ levels with reference to 0 μg/m^3^. The blue line is the estimated exposure–response function. The pink lines demonstrate the first derivatives of the function, indicating the marginal hazard ratio. Panel (b) presents the temporal trends of dust PM_2.5_ exposure and disease burden in different regions in 2000–2017. Light-coloured bars represent the total U5M, whereas dark-coloured bars represent the U5M numbers associated with dust PM_2.5_ exposure with reference to the left y-axis. The line represents the dust PM_2.5_ concentration with reference to the right y-axis. Panel (c) presents the spatial distribution of U5M attributable to life-course dust PM_2.5_ exposure in 2000 and 2017. Review drawing number: GS 京 (2025) 1073号.

The fixed-effects model estimated the dust PM_2.5_–U5M association based on within-strata variations in dust PM_2.5_ exposure. As a result, the linear estimates represent the effects of average dust exposure on child mortality. We employed an optimized method for estimating the exposure–response function to explore how the marginal effects of dust PM_2.5_ varied across exposure levels. In regions with dust PM_2.5_ concentrations below 30 μg/m^3^, point estimates for the marginal effects on U5M were high with wide CIs, indicating a lack of conclusive evidence regarding the threshold concentration. Conversely, in areas with high pollution (>50 μg/m^3^), a significant positive correlation was observed between dust PM_2.5_ and U5M (Fig. 3a).

### Burden of U5M attributable to dust PM_2.5_

Based on the nonlinear exposure–response function without TMREL, we assessed the burden of U5M attributable to dust PM_2.5_ in LMICs. In the analysed 53 LMICs, U5M attributable to dust PM_2.5_ decreased from 1.44 million (95% CI: 0.20–2.46 million) in 2000 to 0.90 million (95% CI: 0.15–1.52 million) in 2017 (Fig. S2). The dust PM_2.5_ exposure levels in these 53 LMICs were similar to those in the 100 LMICs with available baseline U5M data (Fig. S3), with comparable distributions (mean = 14.7 μg/m^3^; SD = 15.45 μg/m^3^ vs. mean = 14.88 μg/m^3^; SD = 15.92 μg/m^3^). Therefore, we extrapolated the nonlinear function to the full set of 100 LMICs to estimate the broader U5M burden (Fig. 3b).

From 2000 to 2017, baseline under-five deaths decreased significantly, dropping from 8.92 million to 5.00 million across 100 LMICs. However, there was no significant improvement in dust pollution, with the population-weighted average of dust PM_2.5_ concentration fluctuating between 15 and 20 μg/m^3^. In 2000, 2010, and 2017, the estimated numbers of U5Ms attributable to dust PM_2.5_ exposure were 1.74 million (95% CI: 0.24–3.00), 1.30 million (95% CI: 0.13–2.28), and 1.07 million (95% CI: 0.17–1.81), accounting for 19.5% (95% CI: 2.7–33.4), 19.4% (95% CI: 1.9–33.8), and 21.3% (95% CI: 3.3–35.6) of the total U5Ms, respectively. Figure 3c presents the spatial distribution of dust PM_2.5_-related burden on U5M at the grid level. In general, sub-Saharan Africa and Central and South Asia were the burden hotspots, collectively accounting for 92.8% of the dust-associated U5M in 2017. At the country level, the highest number of attributable U5Ms in 2017 was in Nigeria (308 870; 95% CI: 126 971–476 033), followed by India (201 664; 95% CI: 4198–374 769) and Pakistan (86 540; 95% CI: 7024–160 314). Table S4 and Fig. S4 present the country-level estimates.

The global burden of dust-related U5M was influenced by the exposure level, demographics, and baseline child mortality rate. Figure S5 presents the distribution of the under-five population, total U5M, and dust-related U5M across different exposure levels in 2000 and 2017. In 2017, in sub-Saharan Africa, the total number of children aged <5 years increased from 110.1 million to 167.3 million, with 14.6% of children residing in heavily polluted areas with dust PM_2.5_ concentrations exceeding 50 μg/m^3^. Conversely, the total number of children in Asian, Latin American, Pacific, and Caribbean regions was significantly reduced, and only 1.5% of children were exposed to high-pollution areas (>50 μg/m^3^). Additionally, East Asia was the only region where the dust concentration showed a decreasing trend, with the attributable fraction reducing from 8.5% to 6.4% (Fig. S6). Our findings indicate that, due to persistently high levels of dust PM_2.5_ pollution and high baseline child mortality, sub-Saharan Africa should be a focal point of concern for dust-related U5M in the future.

**Figure 4.**
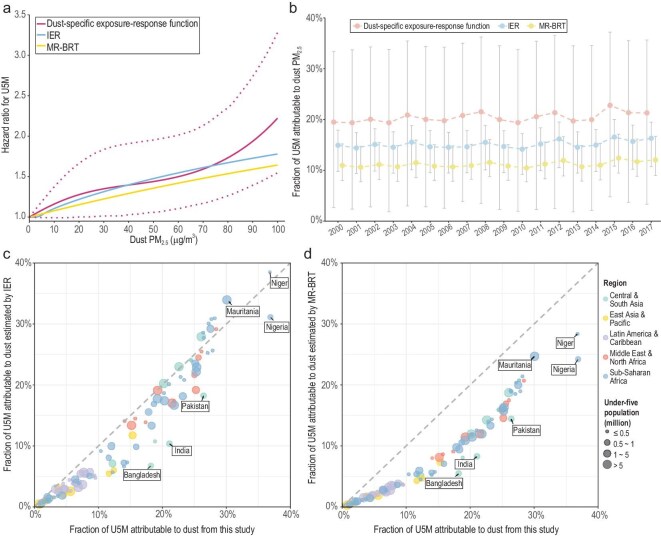
Comparison of the dust-specific exposure–response function in this study in LMICs with IER and MR-BRT. Panel (a) presents three models of the nonlinear exposure–response function for life-course dust PM_2.5_ exposure and U5M. Panel (b) presents the temporal trends of attributable fractions between 2000 and 2017. Panels (c) and (d) present a comparison of country-level attributable fractions among our nonlinear model, IER, and MR-BRT.

Finally, we compared the dust-specific exposure–response function derived from our study with those obtained from the GBD studies (i.e. IER and MR-BRT). Considering the range of uncertainty, these functions were comparable. However, the pre-established functions were sublinear. Conversely, our function was superlinear, which led to different point estimates at high concentrations (Fig. 4a). Based on our function, the attributable fractions for 100 LMICs showed higher point estimates and wider CIs (Fig. 4b). Moreover, for countries with a large number of children, such as India, Pakistan, and Bangladesh, previous exposure–response functions may lead to a considerable underestimation of the dust PM_2.5_ burden on U5M (Fig. 4c, d). These differences highlight the need to establish a dust-specific exposure–response relationship for vulnerable populations.

## DISCUSSION

Based on survey data from LMICs, we explored the association between long-term exposure to dust PM_2.5_ and U5M. The risk of U5M increased by 7.13% (95% CI: 4.54–9.78) for every 10-μg/m^3^ rise in dust PM_2.5_ exposure. The exposure–response function exhibited a nonlinear curve, indicating a high likelihood of no threshold effect at low concentrations and a significant positive association between dust PM_2.5_ and U5M at high concentrations. Using this nonlinear function, we estimated that 21.3% (95% CI: 3.3–35.6) of U5Ms in LMICs in 2017 were attributable to dust PM_2.5_. With a growing population and persistent dust pollution, sub-Saharan Africa, particularly West Africa, has become a hotspot for the related mortality burden. Our findings suggest that previous risk assessment methods without a dust-specific exposure–response function may underestimate the attributable burden of dust PM_2.5_, emphasizing the need to establish a dust-specific exposure–response relationship for vulnerable groups.

Previous studies have reported the adverse effects of PM_2.5_ on child health [22–24]. However, in LMICs, evidence on the association between long-term dust PM_2.5_ exposure and U5M is insufficient. In an analysis of 1 million births with local-level estimates of ambient PM_2.5_ in Africa, Burke *et al.* [20] found that each 10-µg/m^3^ increment in dust PM_2.5_ was associated with a 24% (95% CI: 10–35) increase in infant mortality. Although this point estimate is higher than that in our study, the results are generally comparable given the serious pollution in Africa and increased mortality risk among infants <12 months of age. Additionally, a recent study by Li *et al.* [25] demonstrated that in 88 Asian and African LMICs, 0.48 (95% CI: 0.32–0.64) million premature deaths under the age of 5 years were attributable to windblown dust in 2017. Given the differences in study areas and design, this estimate is in line with our study. Compared to the attributable deaths of outdoor PM_2.5_ exposure reported by the GBD studies [4], our estimates are higher for several reasons. First, our exposure–response function is tailored for children under 5 years of age in LMICs. Given the children's sensitivity to air pollutants, challenging living conditions, and limited access to healthcare in LMICs, the previous mortality burden estimated without accounting for a specific exposure–response function may underestimate the risk of outdoor air pollution. Second, considering that the marginal effects on U5Ms are still considerable in regions with low dust PM_2.5_ concentrations, there was no threshold value in our exposure–response function to estimate premature deaths. Third, the GBD studies primarily used low birth weight, preterm birth, and lower respiratory tract infections as pathways to estimate the mortality burden attributed to long-term PM_2.5_ exposure. If PM_2.5_ is associated with additional adverse outcomes, the health burden would be underestimated.

Several factors contribute to children's vulnerability to dust particles. First, their lungs are still developing, with higher intake of air per unit of body weight compared to adults [7]. The cellular layers within children's respiratory tracts are more permeable, interfering with the biological process of lung development even at low concentrations [26–28]. Second, as the organ with the highest oxygen consumption, the child's brain is in a stage of rapid development. Neurotoxic compounds in air pollution may produce reactions with immune cells in the brain, triggering inflammatory responses and oxidative stress [29,30]. Guxens *et al*. [31] also found an association between PM_2.5_ and thinning of the brain cortex, potentially affecting children's cognitive development. Third, children's bodies are less able to metabolize, detoxify, and excrete the toxicants contained in air pollution. PM_2.5_ deposition in bodies can further contribute to DNA damage and cell cycle arrest [32,33]. Moreover, children live closer to the ground, where some pollutants reach peak concentrations. In summary, children face a higher risk of air pollution, influenced by behavioural, environmental, and physiological factors. Compared to adults, the TMREL for health hazards of dust may be lower or negligible in children.

The Sustainable Development Goals (SDGs) [34] aim to ensure healthy lives for all children and eliminate preventable U5M by 2030. Our findings underscore that the burden of U5M due to dust PM_2.5_ exposure is disproportionately concentrated in sub-Saharan Africa, where frequent dust storms, high birth rates, and limited healthcare access—particularly in neonatal care, postpartum support, and immunization—create a dual challenge. Strengthening early warning systems for dust storms, enhancing the accessibility of child health services, and fostering international cooperation (e.g. South-South collaboration) are essential steps in reducing health inequalities and advancing progress toward the SDGs.

This study has several limitations. First, exposure misclassification was unavoidable due to the confidentiality of respondents’ residential addresses, potential errors in the estimated dust PM_2.5_ concentration, influence of indoor air pollution, and lack of data on migration, activity trajectories of children during pregnancy and early childhood, and gestational length. We also acknowledge the potential issue of immortal time bias. For dust PM_2.5_ exposure, we validated that the concentrations did not exhibit significant time-varying effects, ensuring the reliability of our results (Fig. S7). However, non-dust PM_2.5_ concentrations may have increased over time due to industrialization, leading to an underestimation of the associated health risks. Therefore, the results for non-dust PM_2.5_ should be interpreted with caution. Second, the MERRA-2 dataset was used to extract the monthly dust ratio, its relatively low spatial resolution compared to PM_2.5_ data may result in less precise exposure assessments. Third, our study focused exclusively on live births. Stillbirths resulting from maternal exposure to dust particles during pregnancy were not considered when assessing the impact of dust on children. This may have led to the exclusion of children with better physical health and nutritional status, potentially underestimating the hazards posed by dust exposure. Fourth, the DHS questionnaire relies on interviews with mothers, which may introduce recall bias. To address this, we conducted a sensitivity analysis using a logit regression model (Fig. S8). The logit model showed that the risk of U5M increased by 9.12% (95% CI = 7.82–10.43) for each 10-μg/m³ increase in dust PM_2.5_ concentration, a result statistically comparable to the estimate from the Cox model (7.13%, 95% CI = 4.54–9.78). Fifth, as fixed effects only controlled for unmeasured confounders at the strata level, the existence of residual confounders (e.g. individual socioeconomic status) varying within a stratum could not be completely avoided by covariate adjustment. Finally, our assessment was restricted to 100 LMICs for which the relevant input data was available. Therefore, our results should be interpreted with caution, particularly with regard to generalizability on a global scale.

In conclusion, long-term exposure to dust PM_2.5_ significantly increases the risk of U5M. In most LMICs, particularly in sub-Saharan Africa, dust PM_2.5_ exposure makes a substantial contribution to the U5M burden. Previous burden assessments without a specific function for dust pollution might underestimate the health hazards on children. Sustained dust cleaning could significantly improve children's health in LMICs.

## METHODS

### Study population

Population data for LMICs were obtained from the Demographic and Health Surveys (DHSs), which are nationally representative surveys employing a two-stage cluster sampling method. Initially, strata were generated for each country by dividing each region into urban and rural areas. Clusters were independently selected from each stratum based on the population size. Then, a comprehensive list of households was produced in selected clusters. A fixed or variable number of households were selected using equal probability systematic sampling. To enhance representativeness at the population level, the DHS assigned sample weights within each stratum according to their sampling probabilities. Furthermore, some surveys document the GPS coordinates of respondents’ ‘living clusters’.

In selected households, questionnaires were administered to women aged 15–49 to collect information on marriage, reproductive history, child health, and other related topics. The survival time for each child was recorded with monthly precision. In this survey, U5M refers to deaths occurring between ages 0 and 4 years, including those reported at ages 0 to 59 months and 0 to 99 days.

Up to 1 March 2023, we selected publicly accessible geocoded surveys after DHS-VI that included data on the survival status of children aged <5 years, environmental variables, reproductive conditions, household characteristics, and DHS sampling weights. We excluded data from unreasonable strata, such as small strata that included only respondents from the same living cluster and those that included only cases or controls. Finally, the dataset included 1 411 851 children from 125 surveys, covering 2525 sampling strata distributed across 53 LMICs. Tables S1 and S2 present details of the selected surveys, categorized by year and country, respectively. The number of surveys included each year is relatively consistent.

### Exposure assessment

To determine the monthly average concentration of dust PM_2.5_, we multiplied the PM_2.5_ concentration by the corresponding dust ratio. The monthly average PM_2.5_ concentration, obtained from a global product provided by van Donkelaar *et al*. at a high resolution of 1 × 1 km, was generated through a combination of satellite retrievals of aerosol optical depth, chemical transport modelling, and ground-based measurements [35]. The model predicting annual PM_2.5_ levels demonstrates good performance, with cross-validation *R*^2^ (CV-R^2^) values ranging from 0.81 to 0.86 and average root-mean-square errors (RMSEs) between 8.3 and 8.5 μg/m^3^. The monthly average dust ratio was obtained from the second Modern-Era Retrospective Analysis for Research and Applications (MERRA-2) [36], with a spatial resolution of 0.625° × 0.5°, and is publicly available online (https://goldsmr4.gesdisc.eosdis.nasa.gov/opendap/hyrax/MERRA2/). MERRA-2, based on the Goddard Earth Observing System Model Version 5 (GEOS-5), is the first long-term global reanalysis to integrate space-based aerosol observations and model their interactions with other climate processes. Previous studies have shown that this assimilation effectively identifies Saharan dust events and their transport [37]. Additionally, we included monthly average temperature data from ERA5-Land dataset, a reanalysis dataset provided by the European Centre for Medium-Range Weather Forecasts with a high resolution of 0.1° × 0.1°, as a covariate [38].

For each cluster, we aligned environmental variables within its geographic grid using GPS coordinates. The average lifetime exposure to dust PM_2.5_ served as a metric of long-term exposure of the survey participants. We defined the life course as the period from conception to death or the end of follow-up. Due to the lack of gestational length data in most DHS surveys, the start of the life course was set at 10 months before the birth date. Additionally, we utilized dust PM_2.5_ data from various time windows as a metric of exposure in sensitivity analysis.

### Statistical analyses

Based on the sampling method of the DHS program, we employed a fixed-effect Cox model to evaluate the association between life-course dust PM_2.5_ exposure and U5M. The strata generated during the first stage of DHS sampling were treated as fixed effects in the model, effectively controlling for the variation in omitted variables (i.e. economic, medical, and educational levels) between strata. Additionally, the analysis included variables related to the environment (non-dust PM_2.5_ and temperature), demographics (sex and breastfeeding status), pregnancy (caesarean section, place of delivery, antenatal care attendance, singleton birth or not, and nulliparous or not), reproductive history (maternal age and interpregnancy interval), maternal conditions (BMI and employment status), and household features (sex and age of household head, source of drinking water, and types of toilet and cooking energy). To address the potential nonlinear effects of temperature, maternal age, interpregnancy interval, and age of the household head, these variables were modelled as spline functions with three degrees of freedom. Furthermore, adjustments were made for the seasonal trend in U5M by incorporating interactions between the climate zone of the survey location and the month of child death. DHS sampling weights were also included to ensure the representativeness of results. The regression model can be presented as follows:


(1) \begin{eqnarray*}\begin{array}{@{}*{1}{l}@{}}
{{{\mathrm{h}}}_{{\mathrm{i}},{\mathrm{j}}}( {\mathrm{t}} ) = {{\mathrm{h}}}_{0,{\mathrm{i}}}( {\mathrm{t}})\exp ( {{{\mathrm{x}}}_{{\mathrm{i}},{\mathrm{j}}}\beta + {{\mathrm{z}}}_{{\mathrm{i}},{\mathrm{j}}}\gamma } )}\\ = { {{\mathrm{h}}}_0 ( {\mathrm{t}} )\exp ( {{{\mathrm{x}}}_{{\mathrm{i}},{\mathrm{j}}}\beta + {{\mathrm{z}}}_{{\mathrm{i}},{\mathrm{j}}}\gamma + {\theta }_{\mathrm{i}}} ),}\\ {{\theta }_{\mathrm{i}} = \log [ {{{\mathrm{h}}}_{0,{\mathrm{i}}}( {\mathrm{t}})/{{\mathrm{h}}}_0( {\mathrm{t}} )} ] \ldots \ldots } \end{array}\end{eqnarray*}

where *i* and *j* denote indexes for strata and study subject, respectively; *t* is the survival time (measured by age in months), which equals to the length of lifespan for the U5M cases, or is censored for the under-five children who were alive at the survey date; *h_i_,_j_* is the hazard for *j*th subject of the *i*th stratum; *h_0, i_(t)* is the specific baseline hazard for the *i*th strata; *x_i_,_j_* represents the dust PM_2.5_ exposure; *z_i_,_j_* indicates the adjusted covariates; *β* and *γ* are the corresponding regression coefficients; and *θ_i_* is the fixed effect, representing the average health effect of the sampling center. To further explore the nonlinear exposure–response function, we substituted *x_i_,_j_β* into function *f(x)* in equation (1):


(2) \begin{equation*}{{\mathrm{h}}}_{{\mathrm{i}},{\mathrm{j}}}( {\mathrm{t}} ) = {{\mathrm{h}}}_0( {\mathrm{t}} )\exp [ {{\mathrm{f}}( {{{\mathrm{x}}}_{{\mathrm{i}},{\mathrm{j}}}} ) + {{\mathrm{z}}}_{{\mathrm{i}},{\mathrm{j}}}\gamma + {\theta }_{\mathrm{i}}} ] \ldots \ldots \end{equation*}

The within-strata variation in dust PM_2.5_ exposure was used to determine the health effects on U5M, which represents the marginal change (*Δx_ij_*) in exposure. Therefore, we approximated the function *f(x)* and replaced it with its first Taylor polynomial. Here, *x_i_* denotes the *i*th strata-level mean exposure, and *f’*(*x_i_*) is the first derivative of the nonlinear exposure–response function.


(3) \begin{equation*}{\mathrm{f}}\left( {{{\mathrm{x}}}_{{\mathrm{i}},{\mathrm{j}}}} \right) = {\mathrm{f}}\left( {{{\mathrm{x}}}_{\mathrm{i}} + \Delta {{\mathrm{x}}}_{{\mathrm{ij}}}} \right) \approx {\mathrm{f}}\text{'}\left( {{{\mathrm{x}}}_{\mathrm{i}}} \right)\Delta {{\mathrm{x}}}_{{\mathrm{ij}}} + {\mathrm{f}}\left( {{{\mathrm{x}}}_{\mathrm{i}}} \right)\ \ldots \ldots \end{equation*}

Using a varying-coefficient regression model, we estimated *f’*(*x_i_*) to determine how marginal effects of within-stratum variation in dust PM_2.5_ concentrations vary with stratum-specific averages. The first derivative curve indicated the TMREL of the exposure–response function. At values less than the TMREL, the theoretical exposure–response function remains flat, consistently equalling zero, known as the threshold effect. The TMREL is examined by the point where the marginal effect becomes zero [*f’*(*x_i_*) = 0], as described in our previous work [39].

### Risk assessment

The burden of dust PM_2.5_ exposure on U5M in 100 LMICs between 2000 and 2017 was quantified by extrapolating the exposure-response function derived from 53 LMICs. To achieve this, we aggregated the annual average dust PM_2.5_ concentration and baseline U5M within each 0.625° × 0.5° grid cell. We then applied the estimated nonlinear exposure–response function to calculate the attributable fraction (AF) and attributable number (AN) of U5Ms for each grid for each year using the following formula:


(4) \begin{equation*}\begin{array}{@{}*{1}{l}@{}} {{\mathrm{A}}{{\mathrm{F}}}_{s,t} = 1--1/\exp [ {f( {{x}_{s,t}})}],}\\
{{\mathrm{A}}{{\mathrm{N}}}_{s,t} = {\mathrm{A}}{{\mathrm{F}}}_{s,t} \times {{\mathrm{N}}}_{s,t} \ldots \ldots } \end{array}\end{equation*}

Here, *s* and *t* denote the spatial grid and calendar year, respectively; *x_s_,_t_* is the annual average value of dust PM_2.5_ of the spatial grid; *f* represents the exposure response function; and N*_s_,_t_* signifies the baseline under-five deaths. Population data were obtained from WorldPop (https://www.worldpop.org/). Baseline U5M data were obtained from a grid product generated by the GBD study team for 99 LMICs between 2000 and 2017 [40]. The original spatial resolution of this product is 5 × 5 km, and it is aggregated into the 0.625° × 0.5° grid to align with the resolution of dust PM_2.5_. For India, the product only provided subnational U5M estimates, which are not suitable for our risk assessment. The GBD studies also reported county-level estimates of U5M in India (2000, 2010, and 2017) [41] and China (1996–2012) [42]. Using linear interpolation, we merged these data sources to calculate the U5M numbers in the 0.625° × 0.5° grid for 100 LMICs between 2000 and 2017. Finally, we compared the attributable fraction of U5Ms between our nonlinear model and two well-developed models for PM_2.5_ (integrated exposure–response [IER] [10] and the meta-regression–Bayesian regularized trimmed model [MR-BRT] [4]).

Statistical analyses were performed using R software (version 4.2.2). Regression models were generated using the R packages *fixest* and *survival.* The nonlinear function was modelled by thin plate splines using the R package *mgcv*.

## Supplementary Material

nwaf279_Supplemental_File

## References

[1] Cohen AJ, Brauer M, Burnett R et al. Estimates and 25-year trends of the global burden of disease attributable to ambient air pollution: an analysis of data from the Global Burden of Diseases Study 2015. Lancet 2017; 389: 1907–18. 10.1016/S0140-6736(17)30505-6 28408086 PMC5439030

[2] Landrigan PJ, Fuller R, Acosta NJR et al. The *Lancet* Commission on pollution and health. Lancet 2018; 391: 462–512. 10.1016/S0140-6736(17)32345-0 29056410

[3] World Health Organization . Ambient (outdoor) air pollution. Geneva: World Health Organization; 2024.

[4] Murray CJL, Aravkin AY, Zheng P et al. Global burden of 87 risk factors in 204 countries and territories, 1990–2019: a systematic analysis for the Global Burden of Disease Study 2019. Lancet 2020; 396: 1223–49. 10.1016/S0140-6736(20)30752-2 33069327 PMC7566194

[5] Oliveira M, Slezakova K, Delerue-Matos C et al. Children environmental exposure to particulate matter and polycyclic aromatic hydrocarbons and biomonitoring in school environments: a review on indoor and outdoor exposure levels, major sources and health impacts. Environ Int 2019; 124: 180–204. 10.1016/j.envint.2018.12.052 30654326

[6] Horne BD, Joy EA, Hofmann MG et al. Short-term elevation of fine particulate matter air pollution and acute lower respiratory infection. Am J Respir Crit Care Med 2018; 198: 759–66. 10.1164/rccm.201709-1883OC 29652174

[7] World Health Organization . Air Pollution and Child Health: Prescribing Clean Air. Geneva: World Health Organization; 2018.

[8] McDuffie EE, Martin RV, Spadaro JV et al. Source sector and fuel contributions to ambient PM_2.5_ and attributable mortality across multiple spatial scales. Nat Commun 2021; 12: 12. 10.1038/s41467-021-23853-y 34127654 PMC8203641

[9] Pateraki S, Asimakopoulos DN, Maggos T et al. Chemical characterization, sources and potential health risk of PM_2.5_ and PM_1_ pollution across the Greater Athens Area. Chemosphere 2020; 241: 11. 10.1016/j.chemosphere.2019.125026 31606570

[10] Burnett RT, Pope CA, Ezzati M et al. An integrated risk function for estimating the global burden of disease attributable to ambient fine particulate matter exposure. Environ Health Perspect 2014; 122: 397–403. 10.1289/ehp.1307049 24518036 PMC3984213

[11] Lim SS, Vos T, Flaxman AD et al. A comparative risk assessment of burden of disease and injury attributable to 67 risk factors and risk factor clusters in 21 regions, 1990–2010: a systematic analysis for the Global Burden of Disease Study 2010. Lancet 2012; 380: 2224–60. 10.1016/S0140-6736(12)61766-8 23245609 PMC4156511

[12] Burnett R, Chen H, Szyszkowicz M et al. Global estimates of mortality associated with long-term exposure to outdoor fine particulate matter. Proc Natl Acad Sci USA 2018; 115: 9592–7. 10.1073/pnas.1803222115 30181279 PMC6156628

[13] Weagle CL, Snider G, Li C et al. Global sources of fine particulate matter: interpretation of PM_2.5_ chemical composition observed by SPARTAN using a global chemical transport model. Environ Sci Technol 2018; 52: 11670–81. 10.1021/acs.est.8b01658 30215246

[14] Lelieveld J, Evans JS, Fnais M et al. The contribution of outdoor air pollution sources to premature mortality on a global scale. Nature 2015; 525: 367–71. 10.1038/nature15371 26381985

[15] Ginoux P, Prospero JM, Gill TE et al. Global-scale attribution of anthropogenic and natural dust sources and their emission rates based on Modis Deep Blue Aerosol products. Rev Geophys 2012; 50: 36. 10.1029/2012RG000388

[16] Bachwenkizi J, Liu C, Meng X et al. Fine particulate matter constituents and infant mortality in Africa: a multicountry study. Environ Int 2021; 156: 106739. 10.1016/j.envint.2021.106739 34217038

[17] Goyal N, Karra M, Canning D. Early-life exposure to ambient fine particulate air pollution and infant mortality: pooled evidence from 43 low- and middle-income countries. Int J Epidemiol 2019; 48: 1125–41. 10.1093/ije/dyz090 31074784

[18] Owili PO, Lien WH, Muga MA et al. The associations between types of ambient PM_2.5_ and under-five and maternal mortality in Africa. Int J Environ Res Public Health 2017; 14: 359. 10.3390/ijerph14040359 28358348 PMC5409560

[19] World Health World Health Organization. WHO Global Air Quality Guidelines: Particulate Matter (PM2.5 and PM10), Ozone, Nitrogen Dioxide, Sulfur Dioxide and Carbon Monoxide. Geneva: World Health Organization; 2021. 34662007

[20] Heft-Neal S, Burney J, Bendavid E et al. Dust pollution from the Sahara and African infant mortality. Nat Sustain 2020; 3: 863–71. 10.1038/s41893-020-0562-1

[21] Heft-Neal S, Burney J, Bendavid E et al. Robust relationship between air quality and infant mortality in Africa. Nature 2018; 559: 254–8. 10.1038/s41586-018-0263-3 29950722

[22] Vohra K, Vodonos A, Schwartz J et al. Global mortality from outdoor fine particle pollution generated by fossil fuel combustion: results from GEOS-Chem. Environ Res 2021; 195: 110754. 10.1016/j.envres.2021.110754 33577774

[23] Karimi B, Shokrinezhad B. Air pollution and mortality among infant and children under five years: a systematic review and meta-analysis. Atmos Pollut Res 2020; 11: 61–70. 10.1016/j.apr.2020.02.006

[24] Lelieveld J, Pozzer A, Pöschl U et al. Loss of life expectancy from air pollution compared to other risk factors: a worldwide perspective. Cardiovasc Res 2020; 116: 1910–7. 10.1093/cvr/cvaa025 32123898 PMC7449554

[25] Li PF, Wu JY, Wang RH et al. Source sectors underlying PM2.5-related deaths among children under 5 years of age in 17 low- and middle-income countries. Environ Int 2023; 172: 107756. 10.1016/j.envint.2023.107756 36669285

[26] Gauderman WJ, Urman R, Avol E et al. Association of improved air quality with lung development in children. N Engl J Med 2015; 372: 905–13. 10.1056/NEJMoa1414123 25738666 PMC4430551

[27] Garcia E, Rice MB, Gold DR. Air pollution and lung function in children. J Allergy Clin Immunol 2021; 148: 1–14. 10.1016/j.jaci.2021.05.006 34238501 PMC8274324

[28] Gehring U, Gruzieva O, Agius RM et al. Air pollution exposure and lung function in children: the ESCAPE Project. Environ Health Perspect 2013; 121: 1357–64. 10.1289/ehp.1306770 24076757 PMC3855518

[29] Block ML, Zecca L, Hong JS. Microglia-mediated neurotoxicity: uncovering the molecular mechanisms. Nat Rev Neurosci 2007; 8: 57–69. 10.1038/nrn2038 17180163

[30] Moulton PV, Yang W. Air pollution, oxidative stress, and Alzheimer's disease. Environ Pub Health 2012; 2012: 472751. 10.1155/2012/472751 PMC3317180 22523504

[31] Guxens M, Lubczynska MJ, Muetzel RL et al. Air pollution exposure during fetal life, brain morphology, and cognitive function in school-age children. Biol Psychiatry 2018; 84: 295–303. 10.1016/j.biopsych.2018.01.016 29530279

[32] Xia T, Korge P, Weiss JN et al. Quinones and aromatic chemical compounds in particulate matter induce mitochondrial dysfunction: implications for ultrafine particle toxicity. Environ Health Perspect 2004; 112: 1347–58. 10.1289/ehp.7167 15471724 PMC1247559

[33] Yang J, Huo TT, Zhang X et al. Oxidative stress and cell cycle arrest induced by short-term exposure to dustfall PM_2.5_ in A549 cells. Environ Sci Pollut Res 2018; 25: 22408–19. 10.1007/s11356-017-0430-3 29098582

[34] United Nations . Sustainable Development Goals. (DHHS publication no.: Report Number)| (GPO o. Document Number)|.

[35] van Donkelaar A, Hammer MS, Bindle L et al. Monthly global estimates of fine particulate matter and their uncertainty. Environ Sci Technol 2021; 55: 15287–300. 10.1021/acs.est.1c05309 34724610

[36] Gelaro R, McCarty W, Suárez MJ et al. The Modern-era Retrospective Analysis for Research and Applications, version 2 (MERRA-2). J Clim 2017; 30: 5419–54. 10.1175/JCLI-D-16-0758.1 PMC6999672 32020988

[37] Buchard V, Randles CA, da Silva AM et al. The MERRA-2 aerosol reanalysis, 1980 onward. Part II: evaluation and case studies. J Clim 2017; 30: 6851–72. 10.1175/JCLI-D-16-0613.1 32908329 PMC7477811

[38] Muñoz-Sabater J, Dutra E, Agustí-Panareda A et al. ERA5-land: a state-of-the-art global reanalysis dataset for land applications. Earth Syst Sci Data 2021; 13: 4349–83. 10.5194/essd-13-4349-2021

[39] Xue T, Wang RH, Tong MK et al. Estimating the exposure-response function between long-term ozone exposure and under-5 mortality in 55 low-income and middle-income countries: a retrospective, multicentre, epidemiological study. Lancet Planet Health 2023; 7: E736–46. 10.1016/S2542-5196(23)00165-1 37673544

[40] Burstein R, Henry NJ, Collison ML et al. Mapping 123 million neonatal, infant and child deaths between 2000 and 2017. Nature 2019; 574: 353–8. 10.1038/s41586-019-1545-0 31619795 PMC6800389

[41] Dandona R, Kumar GA, Henry NJ et al. Subnational mapping of under-5 and neonatal mortality trends in India: the Global Burden of Disease Study 2000–17. Lancet 2020; 395: 1640–58. 10.1016/S0140-6736(20)30471-2 32413293 PMC7262604

[42] Wang YP, Li XH, Zhou MG et al. Under-5 mortality in 2851 Chinese counties, 1996–2012: a subnational assessment of achieving MDG 4 goals in China. Lancet 2016; 387: 273–83. 10.1016/S0140-6736(15)00554-1 26510780 PMC5703217

