# Supplementary material for: Estimating under-five mortality attributable to fine particulate matter brought by wind-blown dust in 100 low- and middle-income countries in 2000–2017
Source: Natl Sci Rev. 2025 Jul 9;12(10):nwaf279. doi: 10.1093/nsr/nwaf279 (PMC12492114; doi:10.1093/nsr/nwaf279)
Supplement: nwaf279_Supplemental_File [file nwaf279_supplemental_file.docx]

***Supplemental materials***


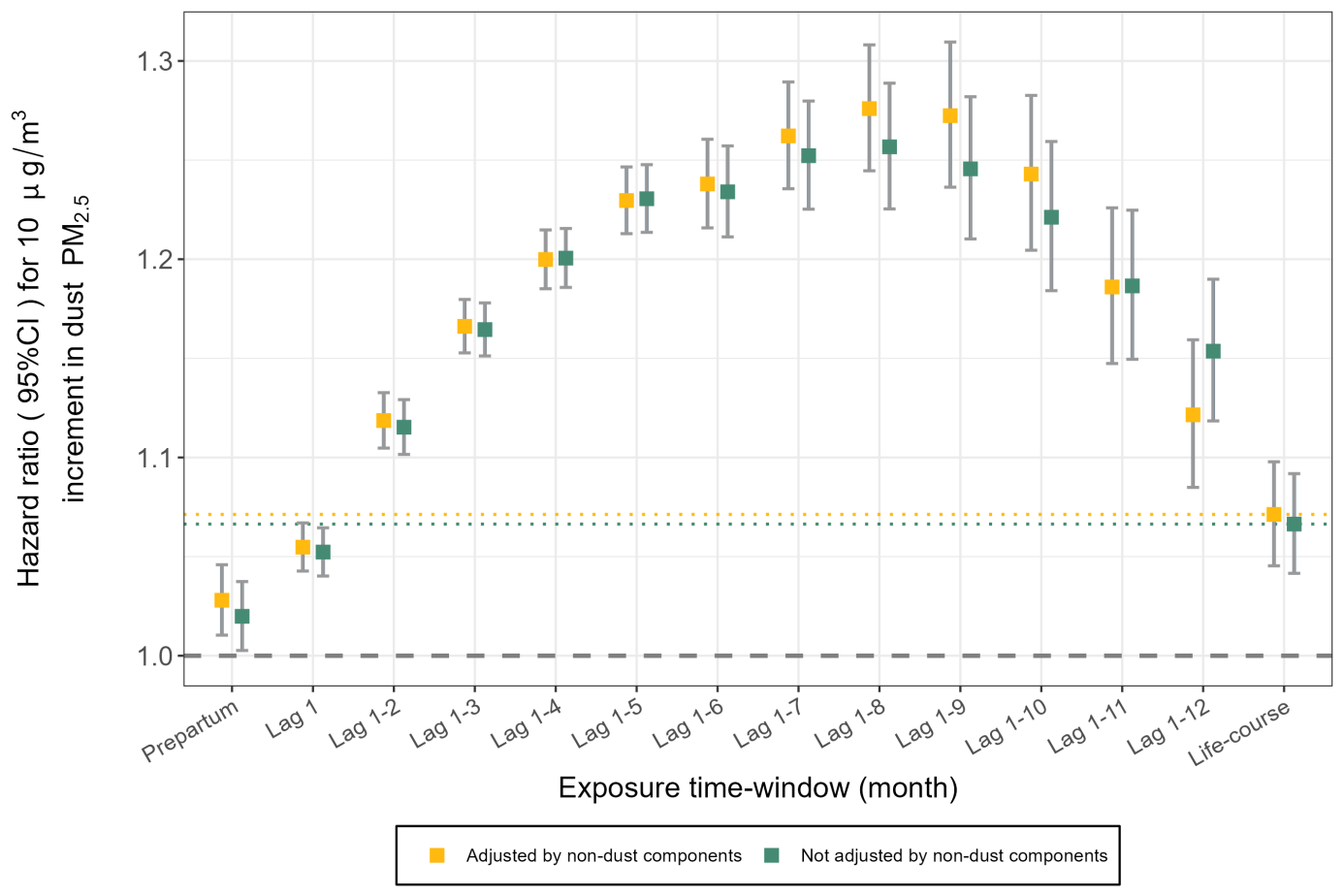


**Figure S1.** Health effects of dust PM_2.5_ on U5M during different exposure time-windows. For the x-axis, *Lag 1-n* denotes the model based on dust concentration in *n* months preceding the outcome, *prepartum* denotes the model for dust exposure during the prenatal period, and *Life-course* denotes our main model.


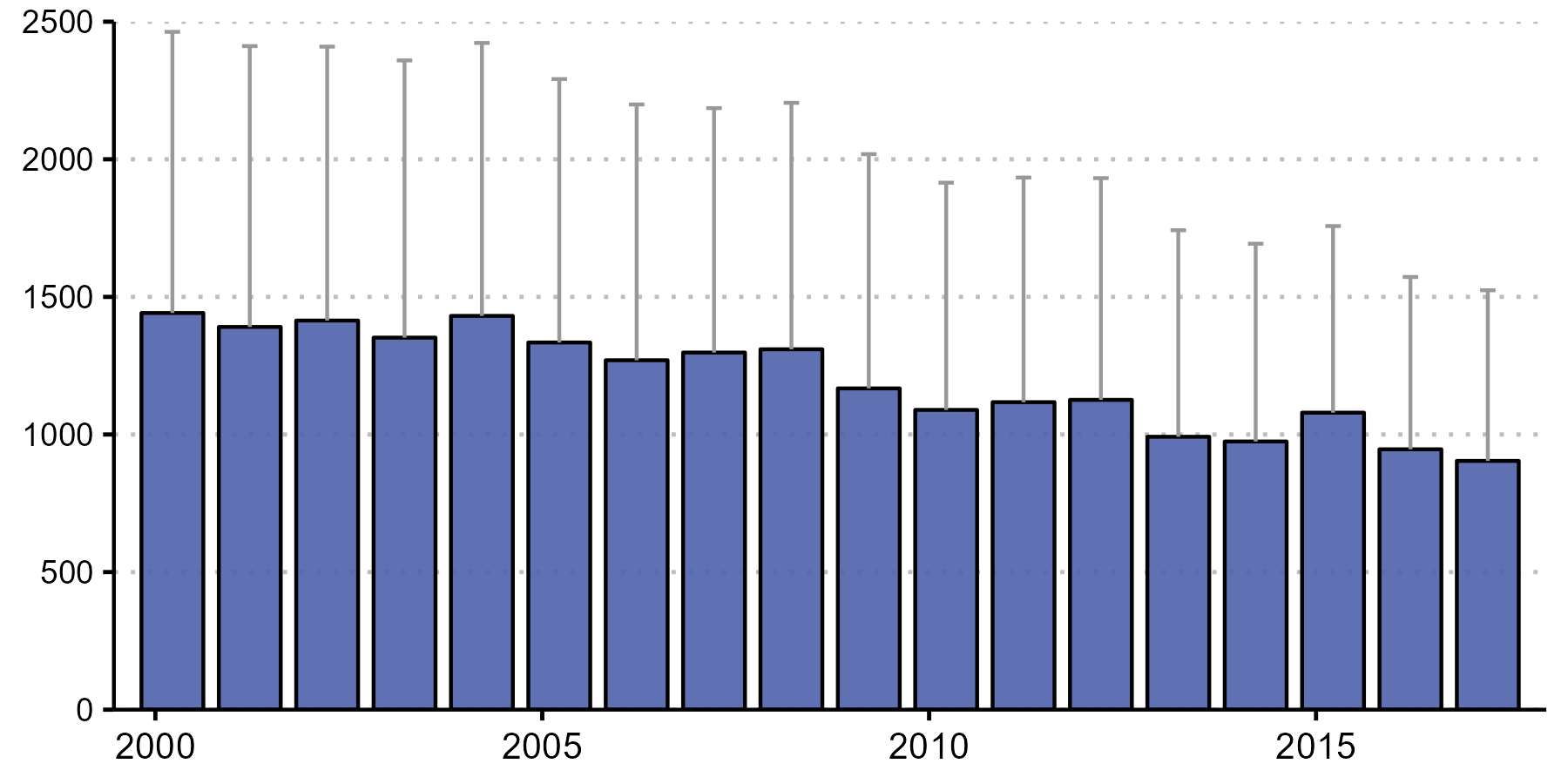


**Figure S2.** Temporal trends of under-five deaths attributable to dust PM_2.5_ exposure in 53 LMICs.


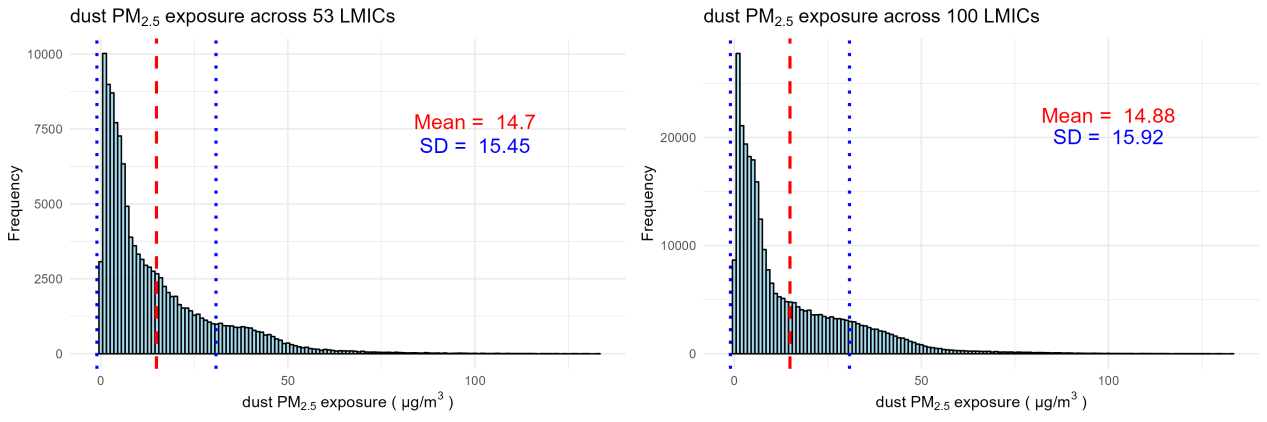


**Figure S3**. Histogram of dust PM_2.5_ exposure levels in LMICs


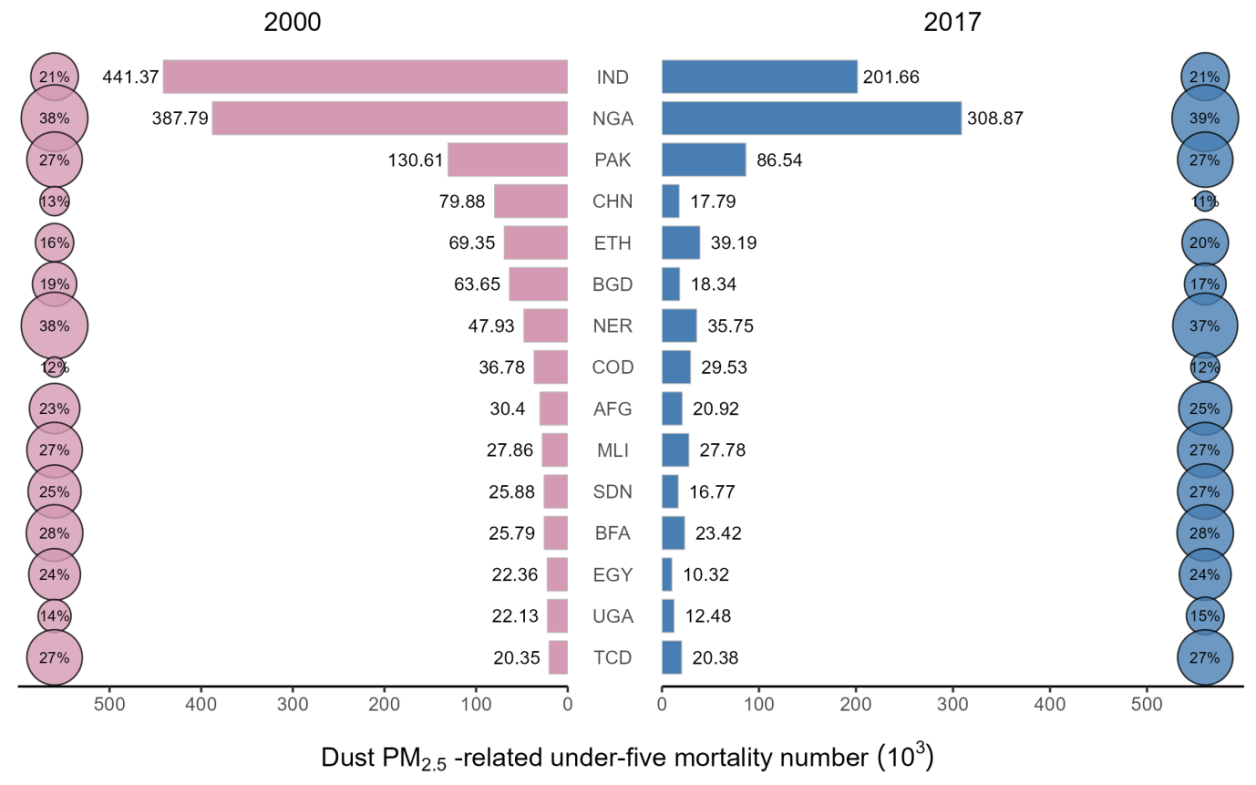


**Figure S4.** Top 15 hotspot countries of dust PM_2.5_ exposure-related U5M burden. Bars indicate the attributed number of deaths, whereas circles represent the U5M fraction attributable to dust PM_2.5_ exposure.


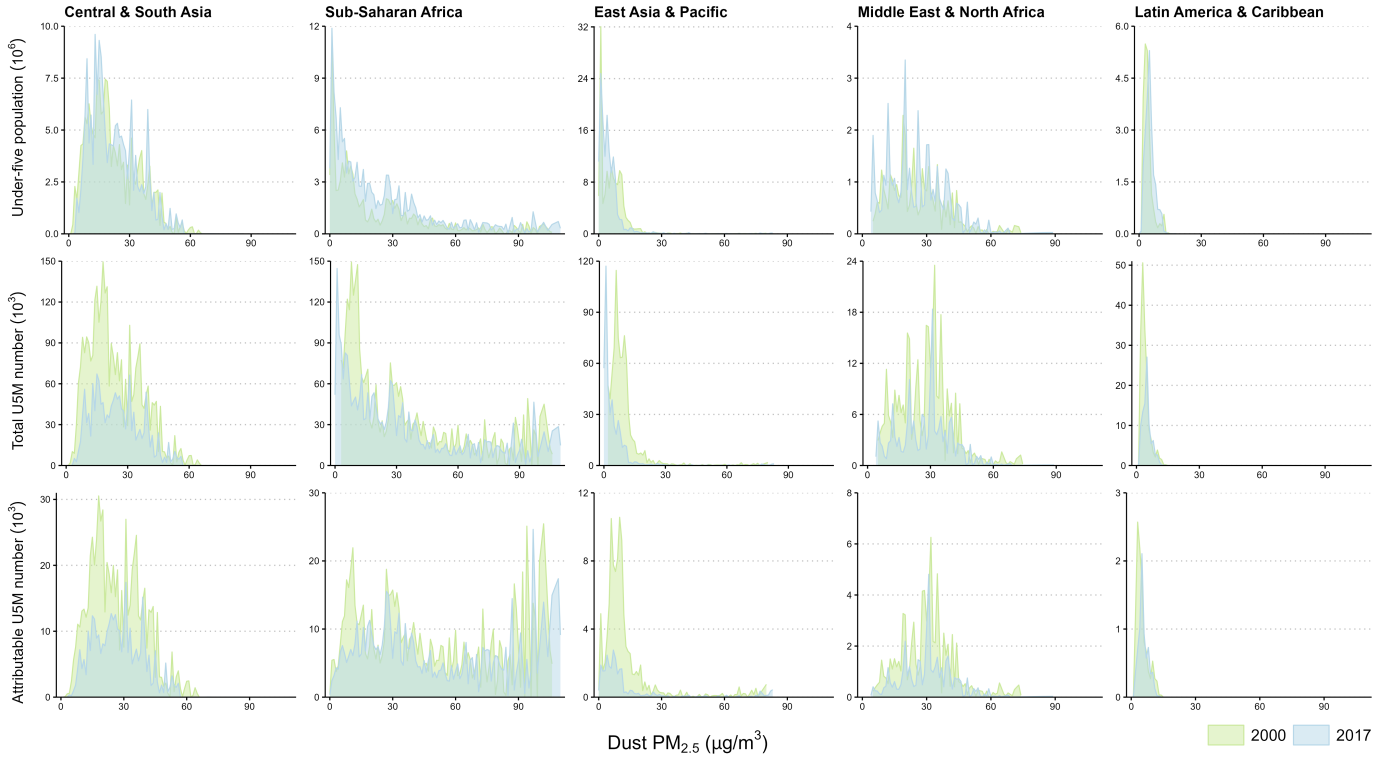


**Figure S5.** Distributions of the under-five exposed population, total under-five deaths, and dust PM_2.5_-related under-five deaths by exposure levels among 100 LMICs in 2000 and 2017. Green represents estimated values for 2000, and blue represents estimated values for 2017.


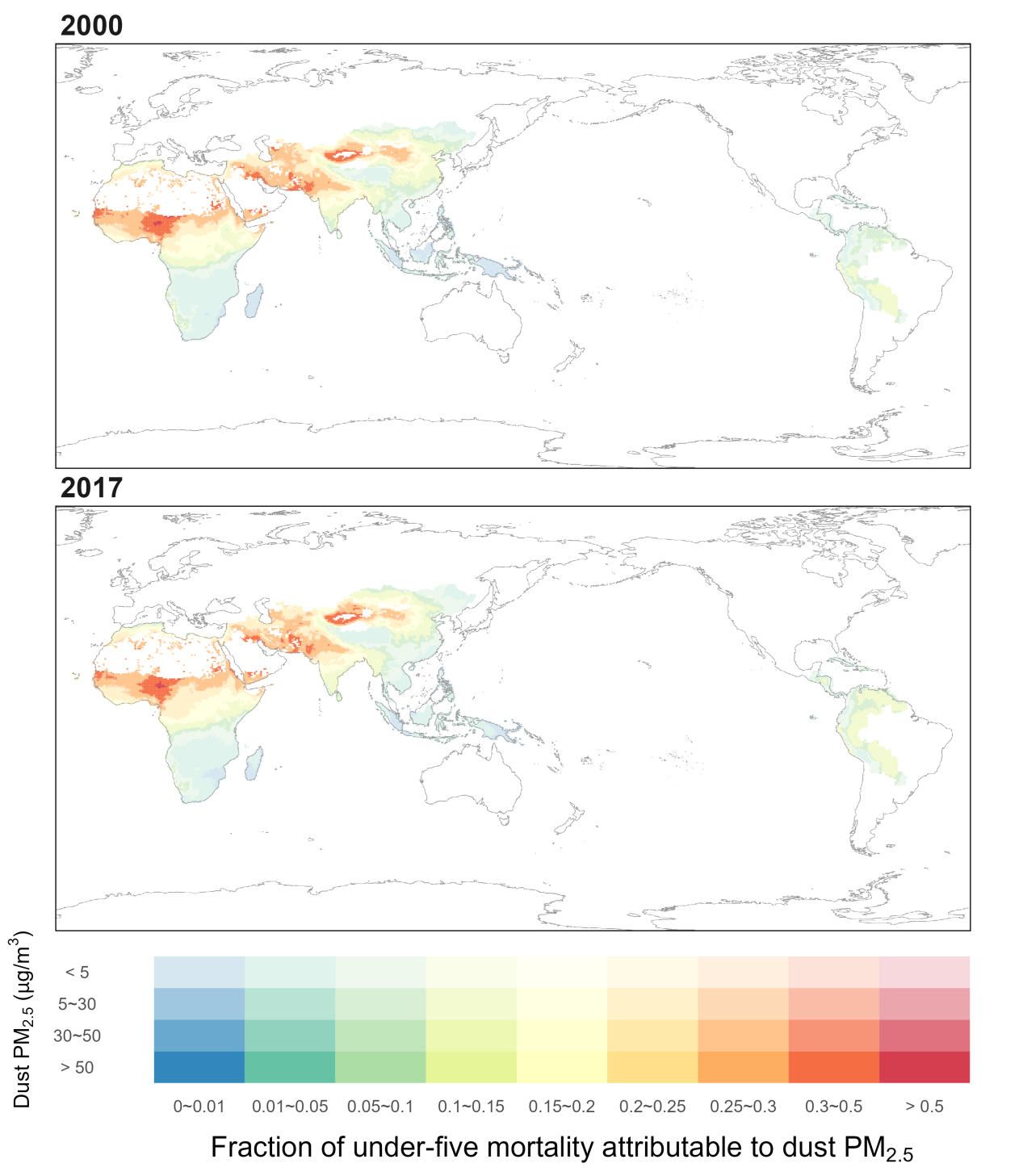


**Figure S6.** Spatial distributions of the attributable fraction of U5M attributable to life-course dust PM_2.5_ exposure in 2000 and 2017, estimated using the nonlinear exposure–response function, in 100 LMICs.


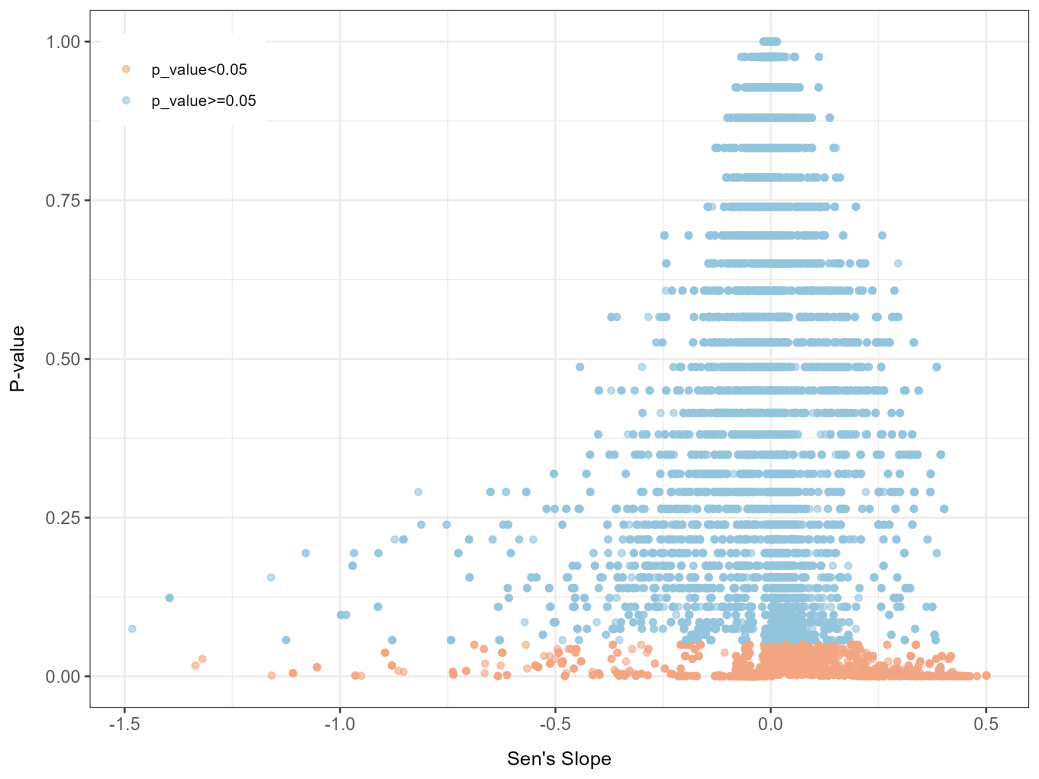


**Figure S7**. Sen's Slope tests for dust PM_2.5_ exposure of clusters included in linear analysis


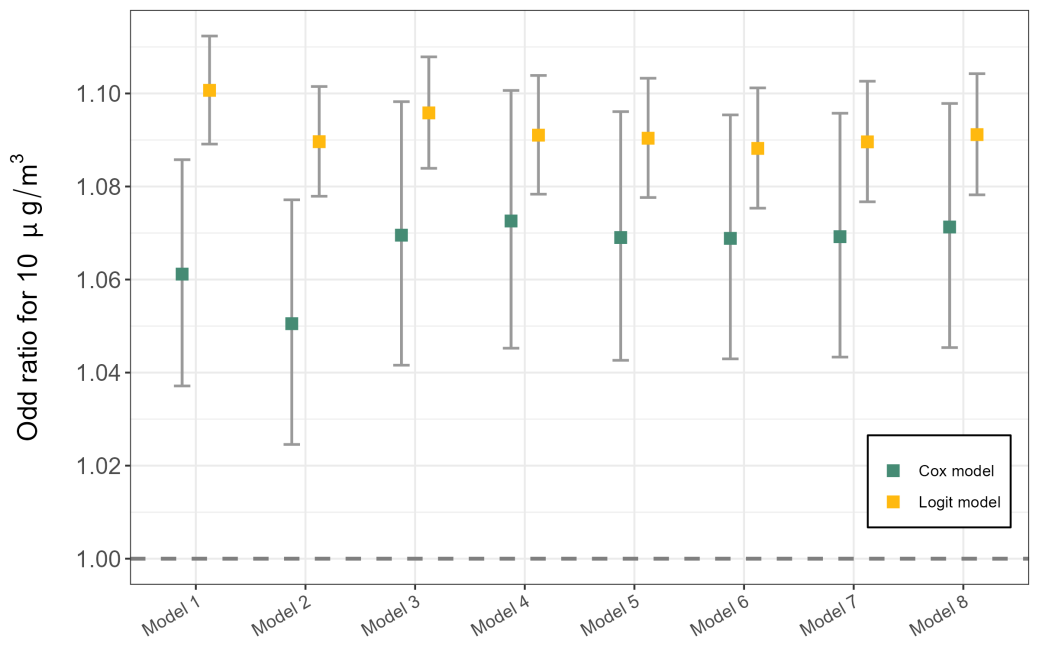


**Figure S8**. Comparison of logit and cox model estimates for linear associations between dust PM_2.5_ exposure and U5M. Model 1: initial model adjusted for the fixed effect of strata; Model 2: Model 1 plus adjustment for seasonal trend (interaction between the climate zone of the survey location and the seasonal child mortality); Model 3: Model 2 plus environmental variables (non-dust components in PM_2.5_ and temperature); Model 4: Model 3 plus demographic characteristics (sex and breastfeeding status); Model 5: Model 4 plus pregnancy-related variables (caesarean section, place of delivery, antenatal care attendance, singleton birth or not, and nulliparous or not); Model 6: Model 5 plus reproductive history-related variables (maternal age and interpregnancy interval); Model 7: Model 6 plus maternal features (maternal body mass index and employment status); Model 8: Model 7 plus household features (sex and age of household head, source of drinking water, and types of toilet and cooking energy).

**Table S1.** Yearly distribution of survey, strata and sample size

| **Year** | **Number of surveys** | **Number of strata** | **Sample size** |
| --- | --- | --- | --- |
| 2004 | 2 | 21 | 2667 |
| 2005 | 9 | 144 | 47954 |
| 2006 | 11 | 160 | 43092 |
| 2007 | 12 | 223 | 56570 |
| 2008 | 13 | 205 | 83116 |
| 2009 | 12 | 234 | 51615 |
| 2010 | 14 | 247 | 91063 |
| 2011 | 15 | 204 | 78271 |
| 2012 | 14 | 243 | 88643 |
| 2013 | 12 | 190 | 94701 |
| 2014 | 14 | 252 | 98184 |
| 2015 | 12 | 311 | 203096 |
| 2016 | 12 | 278 | 185500 |
| 2017 | 11 | 226 | 61624 |
| 2018 | 11 | 203 | 96029 |
| 2019 | 7 | 106 | 124979 |
| 2020 | 3 | 43 | 2944 |
| 2021 | 1 | 24 | 1803 |

*Note: Some surveys within certain strata span multiple years, leading to potential double-counting in the table.

**Table S2.** LMICs and surveys included in the study

| **Country code** | **Survey** | **Sample size** | **Case** | **Control** |
| --- | --- | --- | --- | --- |
| AL | Albania Standard DHS, 2008-09 | 1590 | 23 | 1567 |
|  | Albania Standard DHS, 2017-18 | 2665 | 7 | 2658 |
| AM | Armenia Standard DHS, 2010 | 1473 | 23 | 1450 |
|  | Armenia Standard DHS, 2015-16 | 1724 | 14 | 1710 |
| AO | Angola MIS, 2006-07 | 1576 | 59 | 1517 |
|  | Angola MIS, 2011 | 7461 | 473 | 6988 |
|  | Angola Standard DHS, 2015-16 | 13988 | 693 | 13295 |
| BD | Bangladesh Standard DHS, 2007 | 5794 | 160 | 5634 |
|  | Bangladesh Standard DHS, 2011 | 8504 | 397 | 8107 |
|  | Bangladesh Standard DHS, 2014 | 7673 | 311 | 7362 |
|  | Bangladesh Standard DHS, 2017-18 | 8584 | 345 | 8239 |
| BF | Burkina Faso Standard DHS, 2010 | 14159 | 1246 | 12913 |
| BJ | Benin Standard DHS, 2011-12 | 12878 | 709 | 12169 |
|  | Benin Standard DHS, 2017-18 | 12921 | 902 | 12019 |
| BU | Burundi MIS, 2010 | 7720 | 510 | 7210 |
|  | Burundi Standard DHS, 2016-17 | 13065 | 716 | 12349 |
| CD | Congo Democratic Republic Standard DHS, 2007 | 8371 | 589 | 7782 |
|  | Congo Democratic Republic Standard DHS, 2013-14 | 17199 | 1326 | 15873 |
| CI | Cote d'Ivoire Standard DHS, 2011-12 | 7477 | 655 | 6822 |
| CM | Cameroon Standard DHS, 2011 | 11608 | 987 | 10621 |
|  | Cameroon Standard DHS, 2018 | 9725 | 648 | 9077 |
| CO | Colombia Standard DHS, 2010 | 16513 | 287 | 16226 |
| DR | Dominican Republic Special DHS, 2013 | 858 | 44 | 814 |
|  | Dominican Republic Standard DHS, 2007 | 10161 | 186 | 9975 |
|  | Dominican Republic Standard DHS, 2013 | 3472 | 106 | 3366 |
| EG | Egypt Standard DHS, 2005 | 12225 | 52 | 12173 |
|  | Egypt Standard DHS, 2008 | 10141 | 201 | 9940 |
|  | Egypt Standard DHS, 2014 | 15126 | 369 | 14757 |
| ET | Ethiopia Interim DHS, 2019 | 5753 | 339 | 5414 |
|  | Ethiopia Standard DHS, 2016 | 10092 | 474 | 9618 |
| GA | Gabon Standard DHS, 2012 | 5504 | 287 | 5217 |
| GH | Ghana Standard DHS, 2008 | 2743 | 149 | 2594 |
|  | Ghana Standard DHS, 2014 | 5490 | 273 | 5217 |
| GN | Guinea Standard DHS, 2005 | 5463 | 82 | 5381 |
|  | Guinea Standard DHS, 2012 | 6883 | 603 | 6280 |
|  | Guinea Standard DHS, 2018 | 7782 | 674 | 7108 |
| GU | Guatemala Standard DHS, 2014-15 | 12266 | 354 | 11912 |
| GY | Guyana Standard DHS, 2009 | 1918 | 59 | 1859 |
| HN | Honduras Standard DHS, 2011-12 | 10191 | 266 | 9925 |
| HT | Haiti Standard DHS, 2005-2006 | 5035 | 122 | 4913 |
|  | Haiti Standard DHS, 2012 | 6783 | 473 | 6310 |
|  | Haiti Standard DHS, 2016-17 | 6078 | 386 | 5692 |
| IA | India Standard DHS, 2015-16 | 256269 | 11794 | 244475 |
|  | India Standard DHS, 2019-21 | 108984 | 7829 | 101155 |
| JO | Jordan Standard DHS, 2007 | 9903 | 103 | 9800 |
|  | Jordan Standard DHS, 2012 | 10080 | 230 | 9850 |
|  | Jordan Standard DHS, 2017-18 | 10403 | 182 | 10221 |
| KE | Kenya Standard DHS, 2008-09 | 5957 | 322 | 5635 |
|  | Kenya Standard DHS, 2014 | 20663 | 862 | 19801 |
| KH | Cambodia Standard DHS, 2005 | 7528 | 116 | 7412 |
|  | Cambodia Standard DHS, 2010 | 8027 | 409 | 7618 |
|  | Cambodia Standard DHS, 2014 | 7032 | 191 | 6841 |
| KM | Comoros Standard DHS, 2012 | 2157 | 94 | 2063 |
| KY | Kyrgyz Republic Standard DHS, 2012 | 4312 | 112 | 4200 |
| LB | Liberia MIS, 2009 | 3795 | 293 | 3502 |
|  | Liberia Standard DHS, 2007 | 4772 | 198 | 4574 |
|  | Liberia Standard DHS, 2013 | 7108 | 509 | 6599 |
|  | Liberia Standard DHS, 2019-20 | 3105 | 422 | 2683 |
| LS | Lesotho Standard DHS, 2004 | 1165 | 2 | 1163 |
|  | Lesotho Standard DHS, 2009 | 3961 | 390 | 3571 |
|  | Lesotho Standard DHS, 2014 | 3138 | 223 | 2915 |
| MD | Madagascar Standard DHS, 2008-09 | 11563 | 576 | 10987 |
| ML | Mali Standard DHS, 2006 | 13091 | 716 | 12375 |
|  | Mali Standard DHS, 2012-13 | 10326 | 744 | 9582 |
|  | Mali Standard DHS, 2018 | 9546 | 644 | 8902 |
| MM | Myanmar Standard DHS, 2015-16 | 4683 | 209 | 4474 |
| MW | Malawi Standard DHS, 2004 | 4166 | 21 | 4145 |
|  | Malawi Standard DHS, 2010 | 19490 | 1558 | 17932 |
|  | Malawi Standard DHS, 2015-16 | 17286 | 824 | 16462 |
| MZ | Mozambique Standard AIS, 2015 | 5014 | 156 | 4858 |
|  | Mozambique Standard DHS, 2011 | 10819 | 791 | 10028 |
| NG | Nigeria MIS, 2010 | 5922 | 597 | 5325 |
|  | Nigeria Standard DHS, 2008 | 28085 | 2759 | 25326 |
|  | Nigeria Standard DHS, 2013 | 31064 | 2855 | 28209 |
|  | Nigeria Standard DHS, 2018 | 33658 | 3195 | 30463 |
| NI | Niger Standard DHS, 2012 | 12558 | 956 | 11602 |
| NM | Namibia Standard DHS, 2006-07 | 4883 | 161 | 4722 |
|  | Namibia Standard DHS, 2013 | 4974 | 225 | 4749 |
| NP | Nepal Standard DHS, 2006 | 5563 | 106 | 5457 |
|  | Nepal Standard DHS, 2011 | 5306 | 252 | 5054 |
|  | Nepal Standard DHS, 2016 | 5038 | 177 | 4861 |
| PE | Peru Continuous DHS, 2009 | 9830 | 228 | 9602 |
| PH | Philippines Standard DHS, 2008 | 5317 | 113 | 5204 |
|  | Philippines Standard DHS, 2017 | 8258 | 191 | 8067 |
| PK | Pakistan Standard DHS, 2006-07 | 8649 | 371 | 8278 |
|  | Pakistan Standard DHS, 2017-18 | 10366 | 604 | 9762 |
| RW | Rwanda Interim DHS, 2007-08 | 5269 | 265 | 5004 |
|  | Rwanda Standard DHS, 2005 | 7747 | 108 | 7639 |
|  | Rwanda Standard DHS, 2010 | 8985 | 517 | 8468 |
|  | Rwanda Standard DHS, 2014-15 | 7856 | 298 | 7558 |
|  | Rwanda Standard DHS, 2019-20 | 2445 | 286 | 2159 |
| SL | Sierra Leone Standard DHS, 2008 | 5209 | 441 | 4768 |
|  | Sierra Leone Standard DHS, 2013 | 11538 | 1276 | 10262 |
|  | Sierra Leone Standard DHS, 2019 | 9169 | 776 | 8393 |
| SN | Senegal Continuous DHS, 2012-13 | 6475 | 306 | 6169 |
|  | Senegal Continuous DHS, 2014-6R | 12727 | 602 | 12125 |
|  | Senegal Continuous DHS, 2014-70 | 6252 | 296 | 5956 |
|  | Senegal Continuous DHS, 2015 | 6583 | 316 | 6267 |
|  | Senegal Continuous DHS, 2016-71 | 6275 | 300 | 5975 |
|  | Senegal Continuous DHS, 2017 | 11231 | 543 | 10688 |
|  | Senegal Continuous DHS, 2018 | 6327 | 256 | 6071 |
|  | Senegal Continuous DHS, 2019 | 5738 | 219 | 5519 |
|  | Senegal MIS, 2008-09 | 14105 | 965 | 13140 |
|  | Senegal Standard DHS, 2005 | 9007 | 74 | 8933 |
|  | Senegal Standard DHS, 2010-11 | 11158 | 645 | 10513 |
| SZ | Eswatini Standard DHS, 2006-07 | 2623 | 135 | 2488 |
| TD | Chad Standard DHS, 2014-15 | 18623 | 1722 | 16901 |
| TG | Togo Standard DHS, 2013-14 | 6647 | 420 | 6227 |
| TJ | Tajikistan Standard DHS, 2012 | 4767 | 169 | 4598 |
|  | Tajikistan Standard DHS, 2017 | 6181 | 175 | 6006 |
| TL | Timor-Leste Standard DHS, 2009-10 | 8763 | 449 | 8314 |
|  | Timor-Leste Standard DHS, 2016 | 6362 | 232 | 6130 |
| TZ | Tanzania Standard AIS, 2007-08 | 6703 | 288 | 6415 |
|  | Tanzania Standard DHS, 2010 | 7204 | 451 | 6753 |
|  | Tanzania Standard DHS, 2015-16 | 9725 | 496 | 9229 |
| UG | Uganda MIS, 2009 | 3852 | 252 | 3600 |
|  | Uganda Standard DHS, 2006 | 7179 | 275 | 6904 |
|  | Uganda Standard DHS, 2011 | 7765 | 519 | 7246 |
|  | Uganda Standard DHS, 2016 | 15202 | 796 | 14406 |
| ZM | Zambia Standard DHS, 2007 | 6138 | 343 | 5795 |
|  | Zambia Standard DHS, 2013-14 | 13412 | 739 | 12673 |
|  | Zambia Standard DHS, 2018 | 9730 | 463 | 9267 |
| ZW | Zimbabwe Standard DHS, 2005-06 | 4929 | 91 | 4838 |
|  | Zimbabwe Standard DHS, 2010-11 | 5372 | 349 | 5023 |
|  | Zimbabwe Standard DHS, 2015 | 6132 | 325 | 5807 |

**Table S3.** Descriptive characteristics of the 1,411,851 sample children

| **Characteristic (n, %)** | **Overall** | **control** | **case** | ***P* value** |
| --- | --- | --- | --- | --- |
| Number of participants (N) | 1,411,851 | 1,334,784 | 77,067 |  |
|  | **Mean (Standard Deviation)** | | |  |
| Dust (μg/m^3^) | 19.51 (17.16) | 19.27 (16.90) | 23.66 (20.68) | <0.001 |
| Non-dust (μg/m^3^) | 20.74 (15.99) | 20.70 (15.90) | 21.27 (17.41) | <0.001 |
| Temperature (K) | 296.88 (4.34) | 296.84 (4.36) | 297.61 (3.89) | <0.001 |
| Age month (month) | 27.87 (17.77) | 29.14 (17.30) | 5.93 (9.44) | <0.001 |
| Maternal age (year) | 26.43 (6.34) | 26.44 (6.31) | 26.33 (6.81) | 0.008 |
| Household head age (year) | 42.34 (14.15) | 42.35 (14.16) | 42.24 (13.99) | 0.035 |
| Birth interval (month) | 39.93 (24.32) | 40.20 (24.30) | 35.29 (24.21) | <0.001 |
|  | **N (Percentage %)** | | |  |
| Sex |  |  |  |  |
| Male | 721,335 (51.1) | 679,157 (50.9) | 42,178 (54.7) | <0.001 |
| Female | 690,516 (48.9) | 655,627 (49.9) | 34,889 (45.3) |  |
| Residence |  |  |  |  |
| Urban | 404,773 (28.7) | 387,196 (29.0) | 17,577 (22.8) | <0.001 |
| Rural | 1,007,078 (71.3) | 947,588 (71.0) | 59,490 (77.2) |  |
| Insurance |  |  |  |  |
| No | 880,033 (62.3) | 826,320 (61.9) | 53,713 (69.7) | <0.001 |
| Yes | 119,854 (8.5) | 114,263 (8.6) | 5,591 (7.3) |  |
| Missing | 411,964 (29.2) | 394,201 (29.5) | 17,763 (23.0) |  |
| Breastfeeding |  |  |  |  |
| No | 67961 (4.8) | 46,563 (3.5) | 21,398 (27.8) | <0.001 |
| Yes | 1,196,909 (84.8) | 1,153,994 (86.5) | 42,915 (55.7) |  |
| Missing | 146,981 (10.4) | 134,227 (10.1) | 12,754 (16.5) |  |
| Cesarean-section |  |  |  |  |
| No | 1,206,205 (85.4) | 1,139,185 (85.3) | 67,020 (87.0) | <0.001 |
| Yes | 138,102 (9.8) | 132,566 (9.9) | 5,536 (7.2) |  |
| Missing | 67,544 (4.8) | 63,033 (4.7) | 4,511 (5.9) |  |
| Delivery place |  |  |  |  |
| Home | 465,073 (32.9) | 434,815 (32.6) | 30,258 (39.3) | <0.001 |
| Hospital | 699,910 (49.6) | 665,914 (49.9) | 33,996 (44.1) |  |
| Other | 5,009 (0.4) | 4,786 (0.4) | 223 (0.3) |  |
| Private | 176,252 (12.5) | 168,837 (12.6) | 7,415 (9.6) |  |
| Missing | 65,607 (4.6) | 60,432 (4.5) | 5,175 (6.7) |  |
| Antenatal care |  |  |  |  |
| No | 836,270 (59.2) | 808,352 (60.6) | 27,918 (36.2) | <0.001 |
| Yes | 124,586 (8.8) | 117,961 (8.8) | 6,625 (8.6) |  |
| Missing | 450,995 (31.9) | 408,471 (30.6) | 42,524 (55.2) |  |
| Twins |  |  |  |  |
| No | 1,372,465 (97.2) | 1,303,114 (97,6) | 69,351 (90.0) | <0.001 |
| Yes | 39,386 (2.8) | 31,670 (2.4) | 7,716 (10.0) |  |
| Nulliparous |  |  |  |  |
| No | 1.023,712 (72.5) | 967,245 (72.5) | 56,467 (73.3) | <0.001 |
| Yes | 388,139 (27.5) | 367,539 (27.5) | 20,600 (26.7) |  |
| Maternal BMI |  |  |  |  |
| (-Inf,18.5] | 137,144 (9.7) | 129,328 (9.7) | 7,816 (10.1) | <0.001 |
| (18.5,25] | 598,341 (42.4) | 565,043 (42.3) | 33,298 (43.2) |  |
| (25,30] | 157,499 (11.2) | 150,049 (11.2) | 7,450 (9.7) |  |
| (30, Inf] | 61,746 (4.4) | 59,044 (4.4) | 2,702 (3.5) |  |
| Missing | 457,121 (32.4) | 431,320 (32.3) | 25,801 (33.5) |  |
| Maternal employ |  |  |  |  |
| No | 406,898 (28.8) | 388,453 (29.1) | 18,445 (23.9) | <0.001 |
| Yes | 602,511 (42.7) | 566,461 (42.4) | 36,050 (46.8) |  |
| Missing | 402,442 (28.5) | 379,870 (28.5) | 22,572 (29.3) |  |
| Household head sex |  |  |  |  |
| Male | 1,174,123 (83.2) | 1,109,526 (83.1) | 64,597 (83.8) | <0.001 |
| Female | 237,728 (16.8) | 225,258 (16.9) | 12,470 (16.2) |  |
| Water |  |  |  |  |
| Bottled | 40,949 (2.9) | 39,812 (3.0) | 1,137 (1.5) | <0.001 |
| Natural | 202,895 (14.4) | 190,125 (14.2) | 12,770 (16.6) |  |
| Other | 10,676 (0.8) | 10,191 (0.8) | 485 (0.6) |  |
| Piped | 472,099 (33.4) | 452,391 (33.9) | 19,708 (25.6) |  |
| Rain | 10,845 (0.8) | 10,374 (0.8) | 471 (0.6) |  |
| Tank | 19,834 (1.4) | 18,907 (1.4) | 927 (1.2) |  |
| Tube | 344,393 (24.4) | 322,015 (24.1) | 22,378 (29.0) |  |
| Well | 243,027 (17.2) | 226,920 (17.0) | 16,107 (20.9) |  |
| Missing | 67,133 (4.8) | 64,049 (4.8) | 3,084 (4.0) |  |
| Toilet |  |  |  |  |
| Composting | 21,715 (1.5) | 20,251 (1.5) | 1,464 (1.9) | <0.001 |
| Flush | 398,401 (28.2) | 383,761 (28.8) | 14,640 (19.0) |  |
| No | 384,247 (27.2) | 358,995 (26.9) | 25,252 (32.8) |  |
| Other | 4,191 (0.3) | 3,984 (0.3) | 207 (0.3) |  |
| Pit | 558,806 (39.6) | 525,710 (39.4) | 33,096 (42.9) |  |
| Missing | 44,491 (3.2) | 42,083 (3.2) | 2,408 (3.1) |  |
| Cooking |  |  |  |  |
| Agricultural crop | 19,063 (1.4) | 18,088 (1.4) | 975 (1.3) | <0.001 |
| Animal dung | 37,723 (2.7) | 35,183 (2.6) | 2,540 (3.3) |  |
| Biogas | 4,149 (0.3) | 3,994 (0.3) | 155 (0.2) |  |
| Charcoal | 134,197 (9.5) | 127,529 (9.6) | 6,668 (8.7) |  |
| Coal, lignite | 32,788 (2.3) | 31,498 (2.4) | 1,290 (1.7) |  |
| Electricity | 32,607 (2.3) | 31,619 (2.4) | 988 (1.3) |  |
| Kerosene | 23,756 (1.7) | 22,662 (1.7) | 1,094 (1.4) |  |
| LPG | 199,076 (14.1) | 192,404 (14.4) | 6,672 (8.7) |  |
| Natural gas | 39,212 (2.8) | 38,243 (2.9) | 969 (1.3) |  |
| Other | 1,967 (0.1) | 1,857 (0.1) | 110 (0.1) |  |
| Straw/shrubs/grass | 67,437 (4.8) | 63,478 (4.8) | 3,959 (5.1) |  |
| Wood | 747,000 (52.9) | 698,464 (52.3) | 48,536 (63.0) |  |
| Missing | 72,876 (5.2) | 69,765 (5.2) | 3,111 (4.0) |  |

**Table S4.** Attributable fractions and deaths in 100 LMICs

| **Country code** | **Year** | **Exposure** | **Attributable fractions (%)** | **Attributable deaths** |
| --- | --- | --- | --- | --- |
| AFG | 2000 | 24.3 | 23.0 (0.5, 41.3) | 30399 (586, 57874) |
|  | 2010 | 28.0 | 24.1 (1.2, 42.9) | 22687 (701, 42405) |
|  | 2017 | 29.5 | 24.6 (1.3, 43.5) | 20917 (789, 40563) |
| AGO | 2000 | 3.1 | 5.5 (-0.4, 11.4) | 6636 (-532, 14126) |
|  | 2010 | 4.3 | 6.9 (-0.5, 14.1) | 6491 (-496, 13863) |
|  | 2017 | 4.2 | 6.3 (-0.5, 12.8) | 4295 (-328, 9310) |
| BDI | 2000 | 6.6 | 10.1 (-0.7, 20.3) | 4054 (-263, 8353) |
|  | 2010 | 6.9 | 10.4 (-0.7, 20.9) | 2869 (-185, 5898) |
|  | 2017 | 6.1 | 9.3 (-0.7, 18.7) | 2412 (-163, 5206) |
| BEN | 2000 | 38.7 | 28.0 (3.0, 46.9) | 7926 (908, 13754) |
|  | 2010 | 28.1 | 24.9 (1.3, 43.8) | 7333 (312, 13262) |
|  | 2017 | 40.5 | 28.2 (3.3, 46.9) | 7119 (858, 12506) |
| BFA | 2000 | 37.8 | 27.8 (2.1, 47.1) | 25790 (2214, 45198) |
|  | 2010 | 35.1 | 27.1 (1.5, 46.4) | 23833 (1736, 42365) |
|  | 2017 | 37.1 | 27.7 (1.9, 46.9) | 23421 (1958, 43147) |
| BGD | 2000 | 16.4 | 19.0 (-0.5, 35.6) | 63649 (-1397, 119944) |
|  | 2010 | 12.7 | 16.0 (-0.8, 30.8) | 28550 (-1351, 55871) |
|  | 2017 | 14.3 | 17.3 (-0.7, 32.8) | 18340 (-715, 36624) |
| BLZ | 2000 | 2.1 | 3.9 (-0.3, 8.2) | 11 (-1, 25) |
|  | 2010 | 1.9 | 3.4 (-0.3, 7.2) | 8 (-1, 17) |
|  | 2017 | 2.8 | 5.0 (-0.4, 10.3) | 11 (-1, 23) |
| BOL | 2000 | 6.4 | 9.3 (-0.6, 18.7) | 1718 (-116, 3525) |
|  | 2010 | 5.3 | 8.1 (-0.6, 16.5) | 889 (-65, 1843) |
|  | 2017 | 6.7 | 9.7 (-0.6, 19.6) | 761 (-50, 1599) |
| BTN | 2000 | 9.5 | 13.2 (-0.8, 26.0) | 247 (-14, 491) |
|  | 2010 | 7.4 | 10.8 (-0.7, 21.7) | 120 (-8, 244) |
|  | 2017 | 7.9 | 11.3 (-0.7, 22.4) | 72 (-4, 146) |
| BWA | 2000 | 1.2 | 2.2 (-0.2, 4.6) | 57 (-5, 124) |
|  | 2010 | 1.4 | 2.4 (-0.2, 5.1) | 36 (-3, 78) |
|  | 2017 | 1.1 | 2.0 (-0.2, 4.3) | 22 (-2, 49) |
| CAF | 2000 | 17.6 | 20.2 (-0.2, 37.5) | 5533 (-91, 10916) |
|  | 2010 | 20.0 | 21.6 (0.1, 39.8) | 6437 (10, 12754) |
|  | 2017 | 20.7 | 22.0 (0.2, 40.3) | 5209 (19, 10876) |
| CHN | 2000 | 9.8 | 13.2 (-0.2, 25.2) | 79877 (-1772, 154432) |
|  | 2010 | 10.0 | 13.8 (0.8, 25.4) | 30664 (1352, 57348) |
|  | 2017 | 7.2 | 11.1 (0.5, 20.8) | 17790 (275, 34222) |
| CIV | 2000 | 18.3 | 21.1 (0.2, 38.7) | 18424 (-80, 34962) |
|  | 2010 | 15.5 | 19.1 (-0.4, 35.9) | 15301 (-375, 29471) |
|  | 2017 | 18.8 | 21.1 (0.1, 39.0) | 13600 (-83, 26603) |
| CMR | 2000 | 38.3 | 27.8 (3.1, 46.3) | 19500 (2245, 33448) |
|  | 2010 | 34.7 | 26.9 (1.6, 45.9) | 18806 (1476, 33340) |
|  | 2017 | 41.4 | 28.8 (4.1, 47.3) | 15274 (2123, 27586) |
| COD | 2000 | 8.4 | 11.7 (-0.7, 23.0) | 36776 (-2086, 75879) |
|  | 2010 | 9.0 | 12.6 (-0.6, 24.5) | 39261 (-1774, 80320) |
|  | 2017 | 8.3 | 12.3 (-0.6, 24.0) | 29530 (-1372, 63142) |
| COG | 2000 | 7.7 | 11.4 (-0.7, 22.5) | 3026 (-192, 6046) |
|  | 2010 | 9.6 | 13.1 (-0.7, 25.7) | 2887 (-145, 5920) |
|  | 2017 | 11.6 | 14.2 (-0.7, 27.6) | 2514 (-102, 5541) |
| COL | 2000 | 3.5 | 5.8 (-0.5, 11.9) | 1345 (-108, 2826) |
|  | 2010 | 3.1 | 5.1 (-0.4, 10.7) | 809 (-66, 1724) |
|  | 2017 | 5.1 | 7.9 (-0.6, 16.2) | 906 (-65, 1962) |
| COM | 2000 | 0.6 | 1.0 (-0.1, 2.2) | 20 (-2, 44) |
|  | 2010 | 1.5 | 2.5 (-0.2, 5.2) | 27 (-2, 62) |
|  | 2017 | 1.5 | 2.6 (-0.2, 5.5) | 19 (-2, 45) |
| CPV | 2000 | 22.7 | 22.2 (0.2, 40.7) | 139 (0, 404) |
|  | 2010 | 10.8 | 13.1 (-0.8, 25.9) | 43 (-2, 132) |
|  | 2017 | 20.8 | 20.7 (-0.2, 38.3) | 48 (-0, 142) |
| CRI | 2000 | 2.2 | 3.8 (-0.3, 7.9) | 35 (-3, 73) |
|  | 2010 | 1.8 | 3.0 (-0.3, 6.4) | 23 (-2, 49) |
|  | 2017 | 3.8 | 6.1 (-0.5, 12.7) | 34 (-3, 75) |
| CUB | 2000 | 2.2 | 3.7 (-0.3, 7.9) | 47 (-4, 109) |
|  | 2010 | 2.1 | 3.5 (-0.3, 7.5) | 27 (-2, 61) |
|  | 2017 | 1.9 | 3.2 (-0.3, 6.9) | 19 (-2, 45) |
| DJI | 2000 | 28.2 | 25.4 (1.1, 44.9) | 456 (19, 860) |
|  | 2010 | 28.1 | 24.9 (0.9, 44.3) | 304 (10, 589) |
|  | 2017 | 32.5 | 26.2 (1.3, 45.9) | 218 (10, 444) |
| DOM | 2000 | 2.5 | 4.3 (-0.4, 8.9) | 488 (-42, 1038) |
|  | 2010 | 2.9 | 5.1 (-0.4, 10.7) | 504 (-41, 1067) |
|  | 2017 | 3.2 | 5.5 (-0.4, 11.3) | 390 (-32, 846) |
| DZA | 2000 | 11.4 | 14.9 (-0.5, 28.2) | 3802 (-116, 8377) |
|  | 2010 | 11.5 | 14.4 (-0.3, 27.4) | 2742 (-79, 5945) |
|  | 2017 | 9.3 | 12.3 (-0.5, 24.0) | 1787 (-71, 4280) |
| ECU | 2000 | 3.7 | 5.7 (-0.5, 11.8) | 621 (-51, 1310) |
|  | 2010 | 3.6 | 5.6 (-0.4, 11.6) | 399 (-31, 840) |
|  | 2017 | 4.0 | 6.3 (-0.5, 13.1) | 340 (-27, 746) |
| EGY | 2000 | 26.0 | 24.4 (1.3, 43.1) | 22356 (750, 42187) |
|  | 2010 | 32.6 | 26.5 (1.6, 45.6) | 18174 (1316, 33594) |
|  | 2017 | 26.9 | 24.2 (1.4, 42.5) | 10317 (490, 20745) |
| ERI | 2000 | 28.1 | 24.8 (1.2, 43.8) | 4040 (144, 7454) |
|  | 2010 | 28.5 | 25.3 (1.3, 44.5) | 3321 (146, 6493) |
|  | 2017 | 32.6 | 26.6 (1.6, 45.7) | 2653 (163, 5418) |
| ETH | 2000 | 12.7 | 16.1 (-0.6, 30.8) | 69349 (-2406, 138028) |
|  | 2010 | 16.4 | 19.1 (-0.3, 35.7) | 53156 (-1125, 104153) |
|  | 2017 | 17.5 | 19.7 (-0.0, 36.6) | 39190 (-582, 81546) |
| GAB | 2000 | 10.1 | 15.3 (-0.6, 29.3) | 468 (-15, 968) |
|  | 2010 | 13.0 | 17.5 (-0.5, 33.2) | 498 (-15, 1003) |
|  | 2017 | 15.5 | 19.4 (-0.1, 36.1) | 418 (-7, 906) |
| GHA | 2000 | 28.5 | 25.3 (1.3, 44.6) | 17384 (698, 31068) |
|  | 2010 | 19.5 | 21.4 (0.2, 39.3) | 12098 (-41, 22522) |
|  | 2017 | 25.5 | 24.0 (0.7, 43.0) | 11185 (243, 20815) |
| GIN | 2000 | 24.1 | 23.6 (0.3, 42.4) | 14347 (247, 26729) |
|  | 2010 | 22.6 | 22.9 (0.2, 41.5) | 10993 (133, 20445) |
|  | 2017 | 23.0 | 23.1 (0.2, 41.8) | 9196 (118, 17725) |
| GMB | 2000 | 41.9 | 28.9 (3.4, 47.9) | 1403 (170, 2369) |
|  | 2010 | 34.0 | 27.2 (1.4, 46.7) | 956 (62, 1679) |
|  | 2017 | 38.7 | 28.3 (2.7, 47.5) | 822 (73, 1486) |
| GNB | 2000 | 32.5 | 26.7 (1.5, 46.3) | 2071 (104, 3667) |
|  | 2010 | 26.9 | 25.0 (0.9, 44.4) | 1462 (48, 2688) |
|  | 2017 | 29.6 | 25.8 (1.2, 45.4) | 1189 (54, 2231) |
| GNQ | 2000 | 22.5 | 21.6 (0.2, 39.7) | 627 (4, 1281) |
|  | 2010 | 23.0 | 22.6 (0.2, 40.9) | 453 (3, 904) |
|  | 2017 | 28.1 | 24.6 (1.0, 43.5) | 356 (11, 760) |
| GTM | 2000 | 3.0 | 5.0 (-0.4, 10.4) | 882 (-75, 1860) |
|  | 2010 | 2.1 | 3.6 (-0.3, 7.4) | 419 (-36, 902) |
|  | 2017 | 3.9 | 6.4 (-0.5, 13.1) | 749 (-59, 1627) |
| GUY | 2000 | 5.0 | 7.9 (-0.6, 16.1) | 54 (-4, 115) |
|  | 2010 | 6.2 | 9.4 (-0.7, 19.0) | 44 (-3, 92) |
|  | 2017 | 6.2 | 9.3 (-0.7, 18.9) | 34 (-2, 73) |
| HND | 2000 | 4.0 | 6.4 (-0.5, 13.2) | 398 (-32, 833) |
|  | 2010 | 3.2 | 5.2 (-0.4, 10.9) | 213 (-18, 461) |
|  | 2017 | 5.7 | 8.7 (-0.6, 17.7) | 277 (-20, 597) |
| HTI | 2000 | 3.0 | 4.8 (-0.4, 10.1) | 1217 (-99, 2595) |
|  | 2010 | 3.8 | 6.1 (-0.5, 12.7) | 2953 (-231, 6250) |
|  | 2017 | 4.0 | 6.6 (-0.5, 13.5) | 1178 (-91, 2509) |
| IDN | 2000 | 0.8 | 1.4 (-0.1, 2.9) | 3585 (-325, 7729) |
|  | 2010 | 0.7 | 1.3 (-0.1, 2.7) | 2128 (-193, 4602) |
|  | 2017 | 0.8 | 1.6 (-0.1, 3.4) | 1728 (-152, 3869) |
| IND | 2000 | 20.2 | 20.8 (0.8, 37.5) | 441367 (8356, 806364) |
|  | 2010 | 19.0 | 20.3 (0.4, 36.8) | 305064 (3634, 563045) |
|  | 2017 | 20.8 | 21.5 (0.8, 38.7) | 201665 (4198, 374769) |
| IRN | 2000 | 30.6 | 25.4 (1.8, 44.2) | 15072 (939, 29818) |
|  | 2010 | 32.4 | 26.2 (2.1, 45.3) | 11366 (819, 22281) |
|  | 2017 | 29.9 | 25.2 (1.9, 43.9) | 5093 (317, 10332) |
| IRQ | 2000 | 42.9 | 28.7 (4.0, 47.2) | 10500 (1487, 18776) |
|  | 2010 | 49.4 | 30.5 (6.3, 48.5) | 9679 (1817, 17021) |
|  | 2017 | 37.5 | 27.3 (2.3, 46.1) | 7989 (743, 14853) |
| JAM | 2000 | 3.8 | 6.2 (-0.5, 12.8) | 65 (-5, 150) |
|  | 2010 | 5.0 | 7.7 (-0.6, 16.0) | 61 (-4, 144) |
|  | 2017 | 4.6 | 7.3 (-0.6, 15.1) | 51 (-4, 120) |
| JOR | 2000 | 18.1 | 21.0 (-0.0, 38.8) | 969 (-9, 1902) |
|  | 2010 | 23.5 | 23.9 (0.5, 42.7) | 1007 (26, 1956) |
|  | 2017 | 17.1 | 20.3 (-0.2, 37.5) | 763 (-11, 1569) |
| KEN | 2000 | 6.3 | 9.6 (-0.6, 19.2) | 9412 (-632, 19255) |
|  | 2010 | 8.5 | 12.3 (-0.7, 24.3) | 9270 (-539, 18593) |
|  | 2017 | 7.4 | 11.0 (-0.7, 21.9) | 6714 (-398, 14506) |
| KGZ | 2000 | 16.2 | 19.4 (-0.3, 36.3) | 986 (-17, 1992) |
|  | 2010 | 16.2 | 19.7 (-0.3, 36.8) | 815 (-14, 1644) |
|  | 2017 | 15.9 | 19.4 (-0.4, 36.3) | 630 (-13, 1344) |
| KHM | 2000 | 1.6 | 2.7 (-0.2, 5.8) | 868 (-76, 1883) |
|  | 2010 | 1.5 | 2.6 (-0.2, 5.5) | 416 (-37, 900) |
|  | 2017 | 1.9 | 3.1 (-0.3, 6.5) | 340 (-29, 760) |
| LAO | 2000 | 4.0 | 6.2 (-0.5, 12.8) | 1185 (-92, 2617) |
|  | 2010 | 2.8 | 4.6 (-0.4, 9.7) | 589 (-49, 1314) |
|  | 2017 | 3.1 | 5.1 (-0.4, 10.5) | 449 (-37, 1020) |
| LBR | 2000 | 14.7 | 18.1 (-0.7, 34.4) | 4413 (-159, 8461) |
|  | 2010 | 14.1 | 17.9 (-0.7, 33.9) | 2784 (-100, 5410) |
|  | 2017 | 16.8 | 19.8 (-0.4, 37.1) | 2505 (-48, 4948) |
| LBY | 2000 | 16.1 | 18.6 (-0.2, 34.8) | 410 (-9, 833) |
|  | 2010 | 23.0 | 22.7 (0.2, 41.1) | 288 (4, 595) |
|  | 2017 | 13.0 | 16.0 (-0.5, 29.9) | 173 (-6, 403) |
| LKA | 2000 | 4.3 | 6.9 (-0.5, 14.0) | 431 (-31, 1592) |
|  | 2010 | 5.6 | 8.7 (-0.6, 17.4) | 365 (-24, 1313) |
|  | 2017 | 6.3 | 9.6 (-0.7, 19.4) | 235 (-17, 831) |
| LSO | 2000 | 1.9 | 3.3 (-0.3, 6.9) | 154 (-13, 329) |
|  | 2010 | 2.8 | 4.7 (-0.4, 9.9) | 150 (-13, 321) |
|  | 2017 | 2.5 | 4.2 (-0.4, 8.9) | 99 (-8, 216) |
| MAR | 2000 | 12.2 | 15.7 (-0.7, 29.9) | 5590 (-202, 11508) |
|  | 2010 | 13.8 | 16.9 (-0.6, 31.9) | 3689 (-103, 7836) |
|  | 2017 | 13.9 | 16.9 (-0.5, 31.6) | 2241 (-58, 5281) |
| MDG | 2000 | 0.3 | 0.5 (-0.0, 1.1) | 372 (-34, 829) |
|  | 2010 | 0.7 | 1.1 (-0.1, 2.4) | 832 (-75, 1851) |
|  | 2017 | 0.6 | 1.0 (-0.1, 2.1) | 720 (-63, 1706) |
| MLI | 2000 | 36.5 | 27.5 (1.6, 46.8) | 27856 (2282, 48457) |
|  | 2010 | 36.9 | 27.5 (1.7, 46.7) | 26981 (2272, 47033) |
|  | 2017 | 35.1 | 27.2 (1.5, 46.5) | 27784 (1925, 51525) |
| MMR | 2000 | 7.5 | 10.9 (-0.7, 21.7) | 13061 (-774, 28441) |
|  | 2010 | 6.3 | 9.7 (-0.7, 19.6) | 5908 (-387, 13070) |
|  | 2017 | 8.3 | 12.3 (-0.7, 24.3) | 4819 (-272, 11100) |
| MNG | 2000 | 10.9 | 13.7 (-0.5, 25.9) | 465 (-14, 963) |
|  | 2010 | 15.6 | 16.2 (-0.3, 30.5) | 397 (-9, 823) |
|  | 2017 | 11.3 | 13.8 (-0.7, 26.8) | 264 (-13, 596) |
| MOZ | 2000 | 0.8 | 1.4 (-0.1, 3.0) | 1817 (-164, 3962) |
|  | 2010 | 1.5 | 2.6 (-0.2, 5.4) | 2459 (-213, 5334) |
|  | 2017 | 1.3 | 2.2 (-0.2, 4.6) | 1675 (-144, 3831) |
| MRT | 2000 | 52.5 | 31.3 (6.6, 49.2) | 2525 (533, 4166) |
|  | 2010 | 47.9 | 30.0 (5.2, 48.5) | 1853 (299, 3113) |
|  | 2017 | 50.3 | 30.5 (5.6, 48.8) | 1437 (253, 2539) |
| MWI | 2000 | 1.2 | 2.0 (-0.2, 4.3) | 1498 (-134, 3203) |
|  | 2010 | 2.4 | 4.0 (-0.3, 8.3) | 1917 (-163, 4080) |
|  | 2017 | 1.8 | 3.0 (-0.3, 6.3) | 1064 (-90, 2327) |
| NAM | 2000 | 4.1 | 6.2 (-0.5, 12.8) | 175 (-13, 370) |
|  | 2010 | 4.7 | 6.9 (-0.5, 14.0) | 170 (-13, 362) |
|  | 2017 | 3.6 | 5.4 (-0.4, 11.3) | 103 (-8, 233) |
| NER | 2000 | 66.5 | 37.8 (15.0, 53.7) | 47925 (18541, 72074) |
|  | 2010 | 64.0 | 35.6 (12.2, 51.9) | 37673 (12542, 58294) |
|  | 2017 | 66.8 | 37.2 (14.4, 53.1) | 35751 (13128, 57620) |
| NGA | 2000 | 57.8 | 38.4 (16.2, 54.0) | 387786 (158305, 575652) |
|  | 2010 | 44.0 | 31.6 (8.6, 48.5) | 292935 (76111, 470472) |
|  | 2017 | 58.0 | 39.1 (17.1, 54.6) | 308871 (126972, 476034) |
| NIC | 2000 | 3.4 | 5.4 (-0.4, 11.2) | 249 (-20, 527) |
|  | 2010 | 2.7 | 4.5 (-0.4, 9.4) | 126 (-11, 272) |
|  | 2017 | 5.4 | 8.2 (-0.6, 16.6) | 169 (-12, 373) |
| NPL | 2000 | 17.2 | 19.8 (-0.3, 36.9) | 14323 (-265, 27221) |
|  | 2010 | 13.8 | 17.5 (-0.7, 33.1) | 7127 (-244, 13725) |
|  | 2017 | 15.5 | 18.7 (-0.5, 35.2) | 4980 (-129, 9730) |
| PAK | 2000 | 36.8 | 27.3 (2.3, 46.1) | 130613 (13025, 229838) |
|  | 2010 | 32.5 | 26.0 (1.7, 45.0) | 107407 (6997, 192737) |
|  | 2017 | 34.4 | 26.6 (1.8, 45.5) | 86541 (7024, 160319) |
| PAN | 2000 | 1.8 | 3.0 (-0.3, 6.3) | 45 (-4, 99) |
|  | 2010 | 1.5 | 2.5 (-0.2, 5.3) | 34 (-3, 75) |
|  | 2017 | 2.9 | 4.8 (-0.4, 10.0) | 58 (-5, 137) |
| PER | 2000 | 7.2 | 9.3 (-0.6, 18.5) | 2245 (-148, 4568) |
|  | 2010 | 6.7 | 8.2 (-0.5, 16.5) | 1231 (-84, 2513) |
|  | 2017 | 7.3 | 9.6 (-0.6, 19.2) | 1078 (-69, 2246) |
| PHL | 2000 | 0.7 | 1.2 (-0.1, 2.5) | 1057 (-97, 2326) |
|  | 2010 | 0.9 | 1.5 (-0.1, 3.3) | 1241 (-111, 2757) |
|  | 2017 | 0.9 | 1.6 (-0.1, 3.5) | 1055 (-90, 2439) |
| PNG | 2000 | 0.2 | 0.4 (-0.0, 0.9) | 50 (-5, 115) |
|  | 2010 | 0.3 | 0.6 (-0.1, 1.3) | 87 (-8, 206) |
|  | 2017 | 0.3 | 0.5 (-0.0, 1.1) | 71 (-6, 183) |
| PRY | 2000 | 5.0 | 8.1 (-0.6, 16.5) | 283 (-20, 595) |
|  | 2010 | 3.8 | 6.4 (-0.5, 13.1) | 162 (-13, 350) |
|  | 2017 | 4.8 | 7.8 (-0.6, 15.8) | 136 (-10, 300) |
| PSE | 2000 | 12.2 | 16.5 (-0.8, 31.7) | 469 (-21, 973) |
|  | 2010 | 16.8 | 20.2 (-0.3, 37.6) | 502 (-7, 1000) |
|  | 2017 | 11.6 | 16.0 (-0.8, 30.8) | 262 (-12, 568) |
| RWA | 2000 | 8.4 | 12.1 (-0.7, 24.1) | 7008 (-423, 13803) |
|  | 2010 | 8.5 | 12.2 (-0.7, 24.1) | 3101 (-182, 6353) |
|  | 2017 | 7.5 | 11.0 (-0.7, 22.1) | 2366 (-144, 4894) |
| SDN | 2000 | 31.6 | 25.3 (1.4, 44.5) | 25883 (1146, 48116) |
|  | 2010 | 35.2 | 26.6 (1.6, 46.0) | 22633 (1379, 42067) |
|  | 2017 | 35.7 | 26.8 (1.5, 46.1) | 16768 (1012, 33237) |
| SEN | 2000 | 47.5 | 29.8 (4.8, 48.4) | 13006 (2011, 21538) |
|  | 2010 | 37.1 | 27.8 (1.8, 47.1) | 8317 (662, 14474) |
|  | 2017 | 41.9 | 28.8 (3.4, 47.8) | 6575 (741, 11570) |
| SLE | 2000 | 18.8 | 20.8 (-0.1, 38.5) | 7808 (-67, 14656) |
|  | 2010 | 17.7 | 20.3 (-0.3, 37.7) | 7506 (-112, 14175) |
|  | 2017 | 18.9 | 21.0 (-0.1, 38.8) | 6257 (-39, 11952) |
| SLV | 2000 | 3.8 | 6.2 (-0.5, 12.9) | 253 (-19, 528) |
|  | 2010 | 2.5 | 4.2 (-0.4, 8.8) | 82 (-7, 174) |
|  | 2017 | 5.2 | 8.2 (-0.6, 16.7) | 117 (-9, 251) |
| SOM | 2000 | 9.4 | 10.1 (-0.5, 20.1) | 6227 (-325, 13317) |
|  | 2010 | 8.7 | 10.6 (-0.6, 21.1) | 6037 (-318, 13183) |
|  | 2017 | 9.3 | 11.2 (-0.6, 21.6) | 4908 (-245, 11805) |
| SSD | 2000 | 16.0 | 18.8 (-0.5, 35.4) | 8481 (-190, 16789) |
|  | 2010 | 17.7 | 20.4 (-0.0, 37.8) | 10042 (-122, 19800) |
|  | 2017 | 16.9 | 20.5 (0.0, 38.0) | 9529 (-108, 20134) |
| STP | 2000 | 9.8 | 13.7 (-0.8, 26.8) | 50 (-3, 107) |
|  | 2010 | 6.6 | 10.0 (-0.7, 20.1) | 21 (-1, 47) |
|  | 2017 | 8.9 | 12.7 (-0.7, 25.1) | 19 (-1, 43) |
| SUR | 2000 | 4.8 | 7.6 (-0.6, 15.6) | 30 (-2, 66) |
|  | 2010 | 4.6 | 7.4 (-0.6, 15.1) | 23 (-2, 50) |
|  | 2017 | 5.6 | 8.6 (-0.6, 17.6) | 22 (-2, 51) |
| SWZ | 2000 | 0.8 | 1.3 (-0.1, 2.8) | 36 (-3, 78) |
|  | 2010 | 0.8 | 1.3 (-0.1, 2.8) | 29 (-3, 65) |
|  | 2017 | 0.5 | 0.9 (-0.1, 2.0) | 11 (-1, 24) |
| SYR | 2000 | 20.8 | 21.6 (0.3, 39.5) | 1997 (7, 4310) |
|  | 2010 | 26.0 | 24.1 (0.9, 42.9) | 1771 (50, 3895) |
|  | 2017 | 20.0 | 21.1 (0.2, 38.9) | 1349 (-4, 3073) |
| TCD | 2000 | 36.8 | 26.9 (2.0, 45.9) | 20346 (1769, 35848) |
|  | 2010 | 32.6 | 25.7 (1.6, 44.8) | 20896 (1202, 37597) |
|  | 2017 | 36.2 | 26.9 (2.0, 45.9) | 20380 (1700, 38204) |
| TGO | 2000 | 33.4 | 26.7 (1.6, 46.1) | 5769 (357, 10134) |
|  | 2010 | 23.3 | 23.1 (0.2, 41.8) | 4685 (82, 8728) |
|  | 2017 | 33.3 | 26.4 (1.6, 45.8) | 3932 (236, 7085) |
| THA | 2000 | 2.9 | 5.3 (-0.4, 11.0) | 1174 (-93, 2739) |
|  | 2010 | 2.5 | 4.6 (-0.4, 9.7) | 477 (-38, 1110) |
|  | 2017 | 3.1 | 5.7 (-0.5, 11.7) | 414 (-32, 987) |
| TJK | 2000 | 19.9 | 21.1 (0.2, 39.0) | 2574 (-12, 5056) |
|  | 2010 | 22.5 | 22.5 (0.2, 40.8) | 2281 (22, 4397) |
|  | 2017 | 25.3 | 23.6 (0.6, 42.4) | 2536 (57, 5088) |
| TKM | 2000 | 33.0 | 26.7 (1.6, 46.1) | 1591 (99, 3144) |
|  | 2010 | 30.9 | 26.0 (1.3, 45.4) | 1092 (49, 2202) |
|  | 2017 | 29.6 | 25.4 (1.2, 44.7) | 702 (27, 1477) |
| TLS | 2000 | 1.7 | 2.9 (-0.3, 6.2) | 80 (-7, 174) |
|  | 2010 | 1.8 | 3.0 (-0.3, 6.4) | 41 (-4, 91) |
|  | 2017 | 2.6 | 4.3 (-0.4, 8.8) | 47 (-4, 111) |
| TTO | 2000 | 4.4 | 7.0 (-0.5, 14.3) | 36 (-3, 73) |
|  | 2010 | 6.4 | 9.6 (-0.7, 19.3) | 35 (-2, 72) |
|  | 2017 | 5.5 | 8.5 (-0.6, 17.1) | 27 (-2, 60) |
| TUN | 2000 | 13.8 | 16.9 (-0.6, 32.3) | 806 (-25, 1674) |
|  | 2010 | 13.6 | 16.7 (-0.6, 31.5) | 497 (-16, 1028) |
|  | 2017 | 8.9 | 12.4 (-0.7, 24.0) | 212 (-11, 497) |
| TZA | 2000 | 4.0 | 6.2 (-0.5, 12.6) | 10355 (-773, 21473) |
|  | 2010 | 5.8 | 8.7 (-0.6, 17.5) | 11620 (-802, 23903) |
|  | 2017 | 4.8 | 7.5 (-0.6, 15.2) | 8517 (-623, 18664) |
| UGA | 2000 | 10.2 | 14.2 (-0.8, 27.6) | 22129 (-1219, 43824) |
|  | 2010 | 12.1 | 15.9 (-0.8, 30.7) | 18531 (-909, 36252) |
|  | 2017 | 10.7 | 14.6 (-0.8, 28.5) | 12477 (-638, 25624) |
| UZB | 2000 | 24.6 | 23.6 (0.3, 42.4) | 5450 (101, 11126) |
|  | 2010 | 26.8 | 24.3 (0.8, 43.5) | 4460 (120, 9120) |
|  | 2017 | 27.2 | 24.5 (1.0, 43.7) | 3469 (101, 7427) |
| VEN | 2000 | 4.7 | 7.5 (-0.6, 15.4) | 897 (-67, 1878) |
|  | 2010 | 6.2 | 9.5 (-0.7, 19.2) | 1061 (-72, 2279) |
|  | 2017 | 6.9 | 10.5 (-0.7, 20.9) | 951 (-60, 2098) |
| VNM | 2000 | 2.6 | 4.4 (-0.4, 9.1) | 1893 (-152, 4051) |
|  | 2010 | 2.4 | 4.3 (-0.4, 8.9) | 1224 (-102, 2658) |
|  | 2017 | 2.4 | 4.2 (-0.4, 8.8) | 814 (-66, 1831) |
| YEM | 2000 | 33.4 | 26.5 (1.5, 46.1) | 18412 (1016, 35168) |
|  | 2010 | 29.7 | 25.3 (1.1, 44.7) | 12495 (469, 24362) |
|  | 2017 | 33.8 | 26.6 (1.5, 46.1) | 11014 (595, 21816) |
| ZAF | 2000 | 1.1 | 1.8 (-0.2, 3.9) | 1461 (-130, 3211) |
|  | 2010 | 1.5 | 2.5 (-0.2, 5.3) | 1439 (-126, 3116) |
|  | 2017 | 1.1 | 1.9 (-0.2, 4.0) | 628 (-54, 1387) |
| ZMB | 2000 | 1.6 | 2.8 (-0.2, 6.0) | 1651 (-142, 3573) |
|  | 2010 | 2.1 | 3.6 (-0.3, 7.6) | 1535 (-135, 3340) |
|  | 2017 | 1.8 | 3.0 (-0.3, 6.5) | 1066 (-89, 2461) |
| ZWE | 2000 | 0.8 | 1.4 (-0.1, 2.9) | 436 (-39, 955) |
|  | 2010 | 1.0 | 1.7 (-0.2, 3.6) | 596 (-55, 1299) |
|  | 2017 | 0.8 | 1.4 (-0.1, 3.1) | 368 (-33, 824) |
